# An Optimization Framework of Multiobjective Artificial Bee Colony Algorithm Based on the MOEA Framework

**DOI:** 10.1155/2018/5865168

**Published:** 2018-11-01

**Authors:** Jiuyuan Huo, Liqun Liu

**Affiliations:** ^1^School of Electronic and Information Engineering, Lanzhou Jiaotong University, Lanzhou 730070, China; ^2^Northwest Institute of Eco-Environment and Resources, Chinese Academy of Sciences, Lanzhou 730000, China; ^3^College of Information Science and Technology, Gansu Agricultural University, Lanzhou 730070, China

## Abstract

The artificial bee colony (ABC) algorithm has become one of the popular optimization metaheuristics and has been proven to perform better than many state-of-the-art algorithms for dealing with complex multiobjective optimization problems. However, the multiobjective artificial bee colony (MOABC) algorithm has not been integrated into the common multiobjective optimization frameworks which provide the integrated environments for understanding, reusing, implementation, and comparison of multiobjective algorithms. Therefore, a unified, flexible, configurable, and user-friendly MOABC algorithm framework is presented which combines a multiobjective ABC algorithm named RMOABC and the multiobjective evolution algorithms (MOEA) framework in this paper. The multiobjective optimization framework aims at the development, experimentation, and study of metaheuristics for solving multiobjective optimization problems. The framework was tested on the Walking Fish Group test suite, and a many-objective water resource planning problem was utilized for verification and application. The experiment's results showed the framework can deal with practical multiobjective optimization problems more effectively and flexibly, can provide comprehensive and reliable parameters sets, and can complete reference, comparison, and analysis tasks among multiple optimization algorithms.

## 1. Introduction

The optimization problems in the real world are multiobjective in nature, which means that the optimal decisions need to be taken in the presence of trade-offs between two or more conflicting objectives. These problems are known as multiobjective optimization problems (MOPs) which can be found in many disciplines such as engineering, transportation, economics, medicine, and bioinformatics [1]. Most of the multiobjective techniques have been designed based on the theories of Pareto Sort [2] and nondominated solutions. Thus, the optimum solution for this kind of problem is not a single solution as in the mono-objective case, but rather a set of solutions known as the Pareto optimal set. This refers to when no element in the set is superior to the others for all the objectives.

By using the multiobjective optimization method, the conflicting objectives in these MOPs can acquire better trade-off, and satisfactory optimization results can be given. Therefore, with the complexity and nonlinearity of objectives and constraints, finding a set of good quality nondominated solutions becomes more challenging, and research of efficient and stable multiobjective optimization algorithms is also one of the key and major directions for scholars to study. Over the last few decades, the metaheuristics algorithms [3] have proven to be effective methods for solving MOPs. Among them, the evolutionary algorithms are very popular and widely used to effectively solve complex real-world MOPs [4]. Some of the most well-known algorithms belong to this class, such as the Nondominated Sorted Genetic Algorithm-II (NSGA-II) [5], Multiobjective *ε*-evolutionary Algorithm based on *ε* Dominance (*ε*-MOEA) [6], and Borg [7].

Nevertheless, the swarm intelligence algorithm [8] inspired by biological information is one important type of metaheuristic algorithms. With its unique advantages and mechanisms, it has become a popular and important field. The main algorithms include the particle swarm optimization (PSO) algorithm [9], ant colony optimization (ACO) algorithm [10], and shuffled frog leaping algorithm (SFLA) [11]. In 2005, Karaboga proposed an artificial bee colony (ABC) algorithm based on the foraging behavior of honeybees [12]. ABC has been demonstrated to have a strong ability to solve optimization problems, and its validity and practicality have been proven [13]. Because of achieving high convergence speed and strong robustness, it has been used in different areas of engineering and seems more suitable for multiobjective optimization. At present, the ABC algorithm and its application research mainly focuses on single-objective optimization. The study of multiobjective optimization has just begun.

However, because the multiobjective optimization needs to cope with real problems, there exists some inconvenience in practical applications. For instance, the multiobjective optimization algorithms are closely related to solving problems which are difficult to apply to other MOPs; a consistent model is needed to regulate and compare optimization strategies of different multiobjective optimization algorithms, and users have difficulty choosing the suitable optimization algorithm for their problems and also need to spend a lot of time learning the algorithms.

In this context, it is necessary to establish a unified, universal, and user-friendly multiobjective optimization framework which can be a valuable tool for understanding the behavior of existing techniques, for codes or modules that reuse existing algorithms, and for helping in the implementation and comparison of algorithms' new ideas. Moreover, researchers have found that focusing on the study of one algorithm has a lot of limitations. If different heuristic algorithms can be effectively referred or integrated with each other, they can handle actual problems or large-scale problems more effectively and more flexibly [14].

Therefore, multiobjective optimization frameworks have been proposed to integrate optimization algorithms, optimization problems, evaluation functions, improvement strategies, adjustment methods, and output of results to provide an integrated environment for users to easily handle optimization problems, such as the jMetal [15], Paradiseo-MOEO [16], and PISA [17]. Among them, the MOEA framework [18] is a powerful and efficient platform which is a free and open source Java library for developing and experimenting with multiobjective evolutionary algorithms (MOEAs) and other general purpose multiobjective optimization algorithms.

However, in these integrated environments for MO algorithms, the multiobjective artificial bee colony (MOABC) algorithm has not been integrated yet, and the MOABC algorithm has been proven in our previous research to perform better than many state-of-the-art MO algorithms [19]. Therefore, a multiobjective ABC algorithm named RMOABC [19] was introduced to integrate with the MOEA framework for providing a flexible and configurable MOABC algorithm framework that is independent of specific problems in this paper.

The remainder of this paper is organized as follows. The related literatures are reviewed in Section 2.  Section 3 provides the background concepts and related technologies of MO and introduces the RMOABC algorithm.  Section 4 described the unified optimization framework for MOABC algorithm based on the MOEA framework. The case study is represented in Section 5. The experiment's settings, results, and corresponding analyses are discussed in Section 6, and finally, the conclusions and future work are drawn in Section 7.

## 2. Literature Review

In the past, methods based on metaheuristics developed by simulating various phenomena in the natural world have proven to be effective methods for solving MOPs [20]. Compared to traditional algorithms, modern heuristics are not tied to a specific problem domain and are not sensitive to the mathematical nature of the problem. They are more suitable for dealing with the practical MOPs. A subfamily of them in particular, the evolutionary algorithms, is now widely used to effectively handle MOPs in the real world [21]. In the mid-1980s, the genetic algorithm (GA) began to be applied to solve MOPs. In 1985, Schaffer [22] proposed a vector evaluation GA which realized the combination of the genetic algorithm and multiobjective optimization problems for the first time. In 1989, Goldberg proposed a new idea for solving MOPs by combining Pareto theory in economics with evolutionary algorithms, and it brought important guidance for the subsequent research on multiobjective optimization algorithms [23]. Subsequently, various multiobjective evolution algorithms (MOEAs) have been proposed, and some of them have been successfully applied in engineering [24]. For instance, Li et al. proposed a new multiobjective evolutionary method based on the differential evolution algorithm (MOEA/D-DE) to solve MOPs with complicated Pareto sets [25].

Since 2001, optimization algorithms based on swarm intelligence inspired by the cooperation mechanism of the biological populations have been developed [8]. Through the cooperation of intelligent individuals, the wisdom of the swarm can achieve breakthroughs beyond the optimal individual. Swarm intelligence algorithms have been successfully applied to handle the optimizing problems with more than one objective. For the multiobjective particle swarm optimization (MOPSO) [26] algorithms, a local search procedure and a flight mechanism that are both based on crowding distance are incorporated into the MOPSO algorithm [27]. Kamble et al. proposed a hybrid PSO-based method to handle the flexible job-shop scheduling problem [28]. Leong et al. integrated a dynamic population strategy within the multiple-swarm MOPSO [29].

Among the swarm intelligence algorithm, due to the high accuracy and satisfactory convergence speed, the ABC algorithm shows a greater advantage in problem representation, solving ability, and parameter adjustment [30]. Because research on the multiobjective ABC algorithm has just begun in recent years, there are relatively few studies on MOABC algorithms and its applications. For instance, Hedayatzadeh et al. designed a multiobjective artificial bee colony (MOABC) based on the Pareto theory and *ε*-domination notion [31]. The performance of Pareto-based MOABC algorithm has been investigated by Akbari et al., and the studies showed that the algorithm could provide competitive performance [32]. Zou et al. presented a multiobjective ABC that utilizes the Pareto-dominance concept and maintains the nondominated solutions in an external archive [33]. And Akbari designed a multiobjective bee swarm optimization algorithm (MOBSO) that can adaptively maintain an external archive of nondominated solutions [34]. Zhang et al. presented a hybrid multiobjective ABC (HMABC) for burdening optimization of copper strip production that solved a two-objective problem of minimizing the total cost of materials and maximizing the amount of waste material thrown into the melting furnace [35]. Luo et al. proposed a multiobjective artificial bee colony optimization method called *ε*-MOABC based on performance indicators to solve multiobjective and many-objective problems [36]. Kishor presented a nondominated sorting based multiobjective artificial bee colony algorithm (NSABC) to solve multiobjective optimization problems [37]. Nseef et al. put forward an adaptive multipopulation artificial bee colony (ABC) algorithm for dynamic optimization problems (DOPs) [38]. In our previous works, a multiobjective artificial bee colony algorithm with regulation operators (RMOABC) which utilizes the mechanisms of adaptive grid and regulation operator was proposed in [19]. The experimental results show that compared with the traditional multiobjective algorithms, these variants of multiobjective ABC can find solutions with competitive convergence and diversity within a shorter period of time.

To effectively integrate different heuristic algorithms to handle MOPs more effectively and flexibly, a number of optimization algorithm frameworks were presented and applied in industrial and other fields. For instance, Choobineh et al. proposed a methodology for management of an industrial plant considering the multiple objective functions of asset management, emission control, and utilization of alternative energy resources [39]. Khalili-Damghani et al. proposed an integrated multiobjective framework for solving multiperiod portfolio project selection problems in the investment managers to make portfolio decisions by maximizing profits and minimizing risks over a multiperiod planning horizon [40]. An evolutionary multiobjective framework for business process optimization was presented by Vergidis et al. to construct feasible business process designs with optimum attribute values such as duration and cost [41]. Charitopoulos and Dua presented a unified framework for model-based multiobjective linear process and energy optimization under uncertainty [42]. Tsai and Chen proposed a simulation-based solution framework for tackling the multiobjective inventory optimization problem to minimize three objective functions [43]. A multiobjective, simulation-based optimization framework was developed by Avci and Selim for supply chain inventory optimization to determine supplier flexibility and safety stock levels [44]. Golding et al. introduced a general framework based on ACO for the identification of optimal strategies for mitigating the impact of regional shocks to the global food production network [45]. And a multiobjective optimization framework for automatic calibration of cellular automata land-use models with multiple dynamic land-use classes was presented by Newland et al. [46].

A number of multiobjective optimization framework for more general purposes have also been developed. For example, jMetal is an object-oriented Java-based framework designed to multiobjective optimization using metaheuristics and is available to people interested in multiobjective optimization [47]. PISA is a C-based framework for multiobjective optimization which is based on separating the algorithm specific part of an optimizer from the application-specific part [17]. A framework for dynamic multiobjective big data optimization, jMetalSP combines the multiobjective optimization features of the jMetal framework with the streaming facilities of the Apache Spark cluster computing system that was presented to solve dynamic multiobjective big data optimization problems [48]. The MOEA framework [18] is a powerful and efficient platform that is a free and open source Java library for developing and experimenting with multiobjective evolutionary algorithms (MOEAs) and other general purpose multiobjective optimization algorithms.

In summary, the research of multiobjective ABC algorithms is still in the initial stage, and the MOABC algorithms are still not implemented in the common multiobjective optimization frameworks. Therefore, this paper focuses on introducing the RMOABC algorithm based on the Pareto dominance theory into the MOEA framework to establish a unified, universal, and user-friendly multiobjective optimization framework for the general optimization purpose.

## 3. Background Concepts and Related Technologies

### 3.1. Pareto Dominate Concepts

Multiobjective optimization often has to minimize/maximize two or more nonlinear objectives at the same time which are in conflict with each other. Thus, the trade-offs decisions should be taken between these objectives. Most of the multiobjective algorithms are proposed based on the Pareto Sort [2, 49] theory, so the optimization result is not usually a single solution but rather a set of solutions named as a Pareto nondominated set.

Generally, a multiobjective optimization problem is to optimize a set of objectives subjected to some equality/inequality constraints. The goal of multiobjective optimization is to guide the optimization process towards the true or approximate Pareto front and to generate a well-distributed Pareto optimal set. The basic concepts of the multiobjective method based on the Pareto theory can be found in [50].

### 3.2. Artificial Bee Colony Algorithm

The artificial bee colony (ABC) algorithm is a meta-heuristic and swarm intelligence algorithm proposed by Karaboga [12]. It is inspired by the foraging behavior of honeybees. Each individual bee is taken as an agent, and the swarm intelligence can be guided by the cooperation among different individuals. For its excellent performance, the ABC algorithm has become an effective means for solving complex nonlinear optimization problems.

The three types of bees—employed bees, onlookers, and scouts—constitute the artificial bee colony in the ABC algorithm. The optimization process is changed to the searching process of the nectar foods. Each position of the nectar source represents a feasible solution for the problem, and the nectar amount from the nectar source corresponds to the quality or fitness of the feasible solution. The evolutionary iterations and global convergence are achieved by the cooperation of the three kinds of bees: (1) employed bees perform local random searches in the areas near their food sources; (2) onlookers make an optimum food source to further evolve in accordance with the specific mechanism; and (3) scouts update the stagnant food source according to the processing mechanism for stagnant solutions.

Overall, the employed bees and onlookers can work together to obtain better food sources through random and targeted searches. When the stagnant number of optimal food source reaches a certain value that is prone to fall into the local search, the scouts will start a new random exploration task for the global search. Thus, through the collaboration of the three kinds of bees, the ABC algorithm can quickly and effectively achieve global convergence.

### 3.3. RMOABC Algorithm

A typical goal in a multiobjective optimization problem is to obtain a set of Pareto optimal solutions. As identified earlier, it is necessary to provide a wide variety among the set of solutions for the decision-maker to choose from. By utilizing the Pareto theory, the original ABC algorithm has been improved and extended to handle the MOPs, and the new algorithm is called the RMOABC algorithm [19]. The RMOABC algorithm adopted two mechanisms, regulation operators and adaptive grid, to improve the accuracy and keep the diversity, respectively. And an external archive is also integrated to maintain the historical values of nondominated solutions found in the evolution process.

In the evolution process of optimization algorithms, it is essential to properly control the exploration and exploitation capabilities of the bees to efficiently find the global optimum for the optimization problem. According to the main update equation (i.e., Equation (1)) of the original ABC algorithm, it can be found that more emphasis is taken on the exploration capability [12]:

(1) $$\begin{array}{c} v_{ij}=x_{ij}+\phi_{ij}\;*\;\left(x_{ij}-x_{kj}\right), \end{array}$$

where *x*_*ij*_ (or *v*_*ij*_) denotes the *j*-th element of *X*_*i*_ (or *V*_*i*_); *j* is a random index; *x*_*k*_ denotes another solution selected randomly from the population; and *ϕ*_*ij*_ is a random number in [−1, 1]. It is well known that the exploration and exploitation capabilities of ABC heavily depend on the control parameters in the updated equation of the bees. Thus, to improve the exploitation capability of the ABC algorithm, Zhu et al. proposed a Gbest-guided artificial bee colony algorithm (GABC) in [51] to replace Equation (1) in the original ABC algorithm to obtain

(2) $$\begin{array}{c} v_{ij}=x_{ij}+\phi_{ij}\;*\;\left(x_{ij}-x_{kj}\right)+\psi_{ij}\;*\;\left(y_{j}-x_{ij}\right), \end{array}$$

where *ψ*_*ij*_ is a random number in [0, 1.5] and *y*_*j*_ is the optimal solution fitness value in the *j*-th dimensional space.

To balance the trade-offs between the exploration and exploitation capabilities of MOABC, we proposed a multiobjective artificial bee colony algorithm with regulation operators (RMOABC) in [19] to dynamically adjust the capabilities of exploration and exploitation in the algorithm's evolution process. The local and global dynamic regulation operators were integrated with the GABC algorithm. The mechanisms are to improve the ability of exploitation and guide the search of candidate solutions based on the information of global optimal solutions. The updated Equation (2) in the GABC algorithm was changed into the following equation:

(3) $$\begin{array}{c} v_{ij}=x_{ij}+k\;*\;\phi_{ij}\;*\;\left(x_{ij}-x_{kj}\right)+r\;*\;\left(y_{j}-x_{ij}\right), \end{array}$$

where the local dynamic regulation operator *k* is set to 0.5+cos^2^((*π* *∗* *i*)/(2 *∗* MFE)), the global dynamic regulation operator *r* is set to 0.5+sin^2^((*π* *∗* *i*)/(2 *∗* MFE)), *i* is the current iteration number, and MFE is the maximum iteration number of algorithms. The details can be found in the literature [19].

In the design of multiobjective algorithms, the external archive is a typical method for maintaining the historical values of nondominated solutions found in the evolution process. The adaptive grid [52] mechanism proposed in the PAES (Pareto Archive Evolutionary Strategy) algorithm was utilized in the RMOABC to produce well-distributed nondominated Pareto solutions set in the external archive. Each nondominated solution can be mapped in a certain location in the grid according to the values of multiobjective functions. The grid can adaptively maintain the distribution of candidate solutions stored in the external archive in a uniform way in the evolution process. Thus, these two mechanisms adopted in the RMOABC algorithm can help the algorithm to quickly achieve global convergence. The details can be found in the literature [19].

### 3.4. MOEA Framework

Researchers of optimization algorithms have consensus that there is NO optimum strategy or algorithm for all of the optimized problems, but there is an effective strategy or algorithm for particular optimization problems. Thus, how to efficiently choose the proper algorithm for the particular optimization problem is a challenge for users. As mentioned above, a unified multiobjective optimization framework is good solution that can help users understand the behavior of existing techniques. And it can reuse the codes or modules in existing algorithms and can facilitate the implementation and comparison of new algorithms.

In multiobjective optimization frameworks, the MOEA framework is an open-source evolutionary computation library for Java that specializes in multiobjective optimization [18]. It is also an extensible framework for rapidly designing, developing, executing, and statistically testing multiobjective evolutionary algorithms (MOEAs). The framework supports a variety of state-of-the-art multiobjective evolutionary algorithms (MOEAs) such as NSGA-II (Nondominated Sorting Genetic Algorithm II), NSGA-III (Nondominated Sorting Genetic Algorithm III), *ε*-MOEA (Multiobjective *ε*-evolutionary Algorithm Based on *ε* Dominance), GDE3 (The Third Evolution Step of Generalized Differential Evolution), MOEA/D (Multiobjective Evolutionary Algorithm Based on Decomposition), PISA (Platform and Programming Language Independent Interface for Search Algorithms), and Borg MOEA. It also includes dozens of analytical test problems such as Zitzler-Deb-Thiele (ZDT), Deb-Thiele-Laumanns-Zitzler (DTLZ), CEC2009 (unconstrained problems), and so on. Thus, it can support the multiobjective optimization algorithm to be tested against a suite of state-of-the-art algorithms across a large collection of test problems. The new problems can be conducted in numerous comparative studies to assess the efficiency, reliability, and controllability of state-of-theart MOEAs.

## 4. The Unified Optimization Framework with MOABC Algorithm

The purpose of this paper is to present a unified optimization framework for the MOABC algorithm (UOF-MOABC), which combines the features of the MOEA framework [18] for multiobjective optimization metaheuristics with the RMOABC algorithm presented in [19]. Based on the advantages of a number of classic and modern state-of-the-art optimization, a wide set of benchmark problems and a set of well-known quality indicators assess the performance of the MO algorithms included in the MOEA framework. The UOF-MOABC can assist in multiobjective optimization research at the development, experimentation, comparison, and study of MOABC for solving multiobjective optimization problems.

### 4.1. System Architecture

The architecture of optimization algorithms should be generic enough to allow much needed flexibility to implement most of the metaheuristic; thus, before establishing the optimization framework, the metaheuristic should be characterized by a common behavior that is shared by all its algorithms.

As shown in Algorithm 1, unity procedures of metaheuristics were concluded by Wu et al. in [53]. This algorithm template is similar to most of the optimization algorithms that are based on the metaheuristic search which can be used to implement popular multiobjective technique and foster code reusability.

The MOEA framework has a number of algorithm templates that were summarized from the behavior of the base metaheuristic. Therefore, developing a particular algorithm only requires implementing the specific methods. And MOEA framework was designed based on the object-oriented architecture of Java that can facilitate the creation of new components and reusing of existing ones.

#### 4.1.1. General Architecture of the MOEA Framework

**Figure 1:**
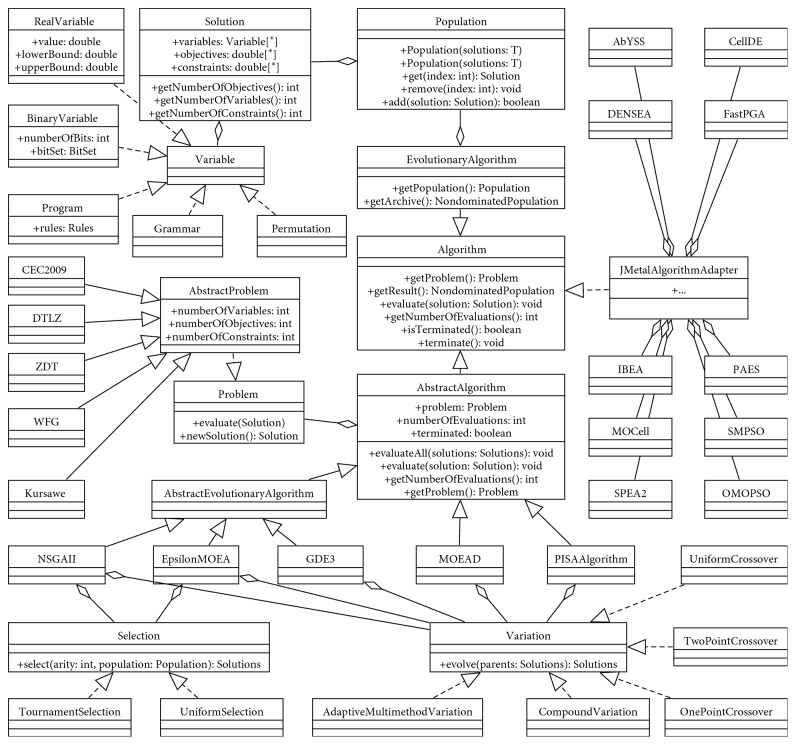
General architecture of the MOEA framework.

The UML diagram, which includes the base classes within the MOEA framework, is summarized and depicted in Figure 1, which was acquired through in-depth analysis and research on the source codes. To make the names of classes more general to be used in most of the metaheuristics, the MOEA framework adopted a generic terminology to name the classes. Therefore, the major components in the MOEA framework include *Algorithm*, *Problem*, and *Solution*. As shown in the figure, the working mechanism of MOEA framework is that an *Algorithm* is adopted to solve a *Problem* using a set of *Solutions*, the *Algorithm* performs the evolution process through a set of *Selection* and *Variation* operations, and the set of nondominated *Solutions* is assessed by the related evaluation methods. In the context of evolutionary algorithms, populations and individuals correspond to *Population* and *Solution* classes in the MOEA framework, respectively. The same classes can be applied to the ABC optimization algorithm related to the concepts of swarm and bees.

The class *Algorithm* represents the superclass for all the optimization metaheuristics. There are two main types of algorithms included in the MOEA framework. One is the native algorithms implemented within the framework that supports all functionality, and the other is the optimization algorithms provided by the JMetal library that can be executed within the MOEA framework. They are represented as the *AbstractAlgorithm* and the *JMetalAlgorithmAdapter* classes which are all inherited the class *Algorithm*, respectively. The classes can combine the optimization algorithm with the *Problem* (getProblem()) and the set of *Solutions* (getResults()) and evaluate the solutions with evaluation methods (evaluation()).

The class *Solution* represents a *Solution* object that is composed of an array of *Variable* objects. The class *Variable* is a superclass aimed at flexibly describing different kinds of representations for solutions that can contain variables of mixed variable types, such as *RealVariable*, *BinaryVariable*, *Program*, *Grammar*, and *Permutation*.

In the MOEA framework, all the problems have to inherit from interface *Problem* and class *AbstractProblem*. The interface *Problem* mainly contains two basic methods: evaluate() and newSolution(). The first method receives a *Solution* object representing a candidate solution to the problem and evaluates it. The second one is to generate a new *Solution* object for the problem according to the algorithm's mechanisms. The *Selection* and *Variation* interfaces aim at representing generic operators to be used by the different algorithms. The *Selection* operators include *TournamentSelection* and *UniformSelection*, and the *Variable* operators include *AdaptiveMultimethodVariation*, *CompoundCrossover*, *OnePointCrossover*, *TwoPointCrossover*, and *UniformCrossover*.

A more detailed description of the MOEA framework can be found in [18] and in the MOEA framework user manual.

#### 4.1.2. Adding MOABC Algorithm to the Framework

**Figure 2:**
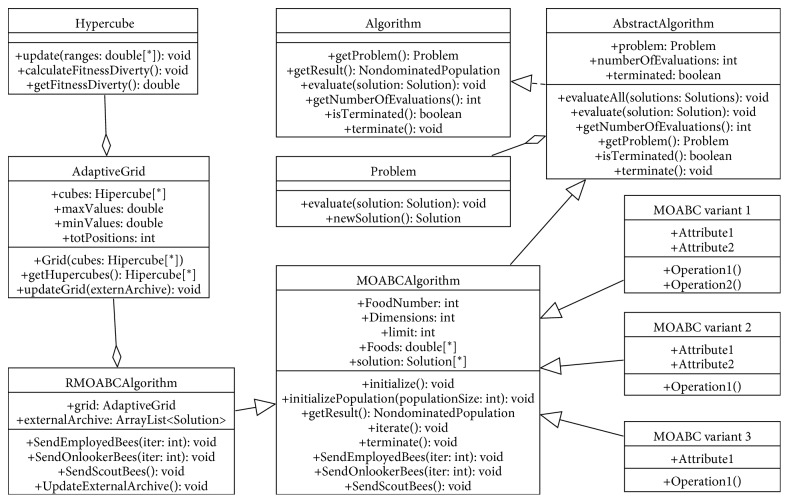
UML diagram of the MOABC algorithm and its variants.

This section is aimed at describing how the MOABC algorithm can be developed and included in the MOEA framework. And the RMOABC algorithm was taken as the demonstration case. To deal with this issue, the new algorithm must be adapted to comply with the standards of the MOEA framework. The UML class diagram of the MOABC algorithm and its variants which were implemented in the MOEA framework is depicted in Figure 2. To make the framework more versatile, we first designed a common class *MOABCAlgorithm* to implement the general attributes and methods of the MOABC algorithm. Through inheriting and implementing methods in the class *AbstractAlgorithm* of the MOEA framework, it achieves the integration of the MOABC algorithm with the MOEA framework. Then, the implementation classes of the MOABC algorithm and its variant algorithms inherit the *MOABCAglorithm* class and implement their own mechanisms by adding or modifying the attributes and methods.

Taking the RMOABC algorithm as a case, the class *RMOABCAlgorithm* needs to inherit the class *MOABCAlgorithm* and implements its specific methods such as SendEmployedBees(), SendOnlookerBees(), SendScoutBees(), and UpdateExternalArchive(). The Adaptive Grid mechanism of RMOABC produces well-distributed nondominated Pareto solutions set in the external archive, which is implemented by class *AdaptiveGrid* and class *Hypercube*.

After implementation of the MOABC algorithm in the MOEA framework, we began to describe how to execute it. The *Executor*, *Instrumenter*, and *Analyzer* classes provide most of the functionality provided by the MOEA framework [18]. The *Executor* class is responsible for constructing and executing runs of an algorithm. A single run requires three pieces of information: (1) the problem; (2) the algorithm used to solve the problem; and (3) the number of objective function evaluations allocated to solve the problem. The *Instrumenter* class works with the *Executor* class to record the necessary data while the algorithm is running. And the *Analyzer* class provides end-of-run analysis which is useful for statistically comparing the results produced by the different algorithms.

### 4.2. General Optimization Framework

**Figure 3:**
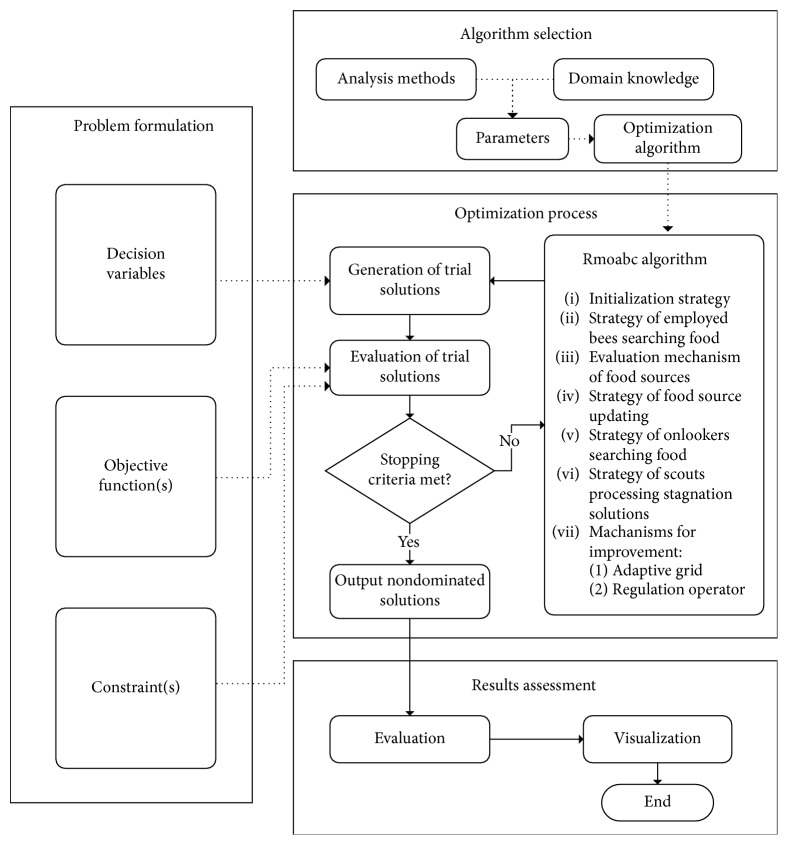
UOF-MOABC general optimization framework.

Figure 3 gives an overview of the proposed UOF-MOABC framework for the general purpose optimization problems. As shown in the figure, the framework is comprised of four stages: *Problem Formulation*, *Algorithm Selection*, *Optimization Process*, and *Result Assessment* for RMOABC algorithm to solve the optimization problems.

#### 4.2.1. Problem Formulation

In the *Problem Formulation* stage, the decision variables in this optimization problem and the specification of the objective function to be optimized, as well as any constraints on the decision variables or solutions need to be formulated according to the standards of the MOEA framework.

*(1) Decision Variables*. In the context of optimization, decision variables are unknown and controllable options that need to be determined in order to solve the problem; in other words, the problem is solved when the best values of the variables have been identified. The values of decision variables determine the values of the objective functions. The defining of decision variables is one of the hardest and most crucial steps in formulating the optimized problem. Sometimes, a creative and suitable definition for the decision variables can dramatically reduce the size and difficulty of the problem. Thus, the MOEA framework provides different kinds of representations such as *RealVariable*, *BinaryVariable*, *Program*, *Grammar*, and *Permutation* for solutions which can flexibly contain variables of mixed variable types.

*(2) Objective Functions*. The objective function of an optimization problem indicates how much each decision variable contributes to the value to be optimized in the problem. As shown in Equation (4), the objective of the optimization process is to minimize or maximize the numerical value of the objective function by changing the values of selected decision variables:

(4) $$\begin{array}{c} \text{objective}\;\;\mathrm{function}=f\left(x_{1},x_{2},\ldots ,x_{i},\ldots x_{n}\right), \end{array}$$

where *x*=(*x*_1_, *x*_2_,…, *x*_*i*_,…*x*_*n*_) is the vector of decision variables and *x*_*i*_ denotes the *i*-th decision variable in the *n*-dimensional decision space.

*(3) Constraints*. Constraints define the possible values the decision variables of an optimization problem may take. They typically represent resource constraints, or the minimum or maximum levels of an activity and take the general form as follows:

(5) $$\begin{array}{c} \text{subject}\;\;\mathrm{to}\;g_{i}\left(x\right)\leq 0,\;j=1,2,\ldots ,J,\\ \;\;\;\;\;h_{k}\left(x\right)=0,\;k=1,2,\ldots ,K, \end{array}$$

where *x*=(*x*_1_, *x*_2_,…, *x*_*i*_,…*x*_*n*_) is the vector of decision variables, *x*_*i*_ denotes the *i*-th decision variable in the *n*-dimensional decision space, *g*_*i*_(*x*) ≤ 0 defines *J* inequality constraints, and *h*_*k*_(*x*)=0 defines *K* equality constraints. Note that *j* is an index that runs from 1 to *J*, and each value of *j* corresponds to an inequality constraint. *k* is an index that runs from 1 to *K*, and each value of *k* corresponds to an equality constraint. The constraints generally reflect limitations of the real world system under consideration.

#### 4.2.2. Algorithm Selection

Once the optimization problem has been formulated correctly, the optimization framework needs to choose the appropriate and suitable optimization algorithm for the problem in the *Algorithm Selection* stage. There are several configurations that need to be specified before running the optimization algorithm. Firstly, the parameters of selected optimization algorithms must be configured based on suggested values from the literature or from previous experiences. These values of parameters influence optimization processes or search behaviors of the algorithm, such as population size, food source number, *Limit* number, and size of external archive in the case of the multiobjective ABC algorithm. Secondly, the termination criteria for the optimization process such as a predefined maximum iterations number or the precision value of no significant improvement in performance, must be specified. And finally, the number of execution times of the entire optimization process that has to be repeated must be specified to eliminate the randomness effects for the solutions, and to increase the chance for the optimal nondominated solutions.

#### 4.2.3. Optimization Process

The purpose of this stage is to use the selected optimization algorithm (such as the RMOABC algorithm) to identify the nondominated solutions for the optimization problem that provide the best possible trade-offs between the selected objective functions. Then, the problem can be solved using the optimization algorithm in the following optimization processes. The solution generation mechanisms are used to generate trial solutions for the optimized problem from the search space of each decision variable. Various strategies can be adopted in the solution generation mechanisms to improve the computational efficiency and accuracy of the solutions. These trial solutions are then evaluated in terms of their constraints to verify whether they violate the constraints of the problem. And objective function values are also evaluated to judge whether the trial solution is better than the previous solution (for single objective problems) or whether it is a nondominated solution to the problem (for multiobjective problem). Then, the algorithm judges whether the stopping criteria have been met, and, if so, the algorithm will output the resulting nondominated solutions; otherwise, information from the previous evaluation process is used to guide the generation of the next trial solutions using an optimization algorithm.

#### 4.2.4. Result Assessment

Finally, in the last *Result Assessment* stage, the nondominated solutions for the optimized problem are quantitatively assessed and visualized. Then, the algorithm finishes the operation.

Many quantitative evaluation methods can be used to evaluate nondominated solutions to the optimal Pareto for the multiobjective optimization algorithms. These can mainly be divided into two categories: convergence and distribution [54]. The convergence indicator denotes the distance between the calculated noninferior front and the known true noninferior front or approximate true noninferior front. The distribution indicator refers to whether the obtained noninferior sets are evenly distributed in the optimal space. The most common used indicators are Generational Distance (GD) [55], Inverted Generational Distance (IGD) [56], Δ_p_ [57], Spacing (SP) [58], Hypervolume (HV) [59], and Computational Time (Times).

## 5. Test Problems

In this section, how the framework can be used for optimizing a practical problem will be discussed in detail. A well-known problem named the water resource planning (WRP) problem in the multiobjective literatures and the Walking Fish Group (WFG) test suite are taken as the test problems.

### 5.1. Water Resources Plan Problem

The water resource planning problem involves optimal planning for a storm drainage system in an urban area that was originally described by Musselman and Talavage in [60]. The problem entails examining a particular subbasin within a watershed. The subbasin is assumed to be hydrologically independent of other subbasins, having its own drainage network, on-site detention storage facility, treatment plant, and tributary to a receiving water body [61]. Mathematically, the WRP problem is a three-variable, five-objective, seven-constraint real-world problem to optimize the planning for a storm drainage system in an urban area. A detailed description of the problem can be found in [61], and it has been implemented in the MOEA framework.

#### 5.1.1. Decision Variables

Three decision variables are assumed to characterize the storm drainage system of the subbasin: **x**_1_ is the local detention storage capacity (unit: basin·inches), **x**_2_ is the maximum treatment rate (unit: basin·inches/hour), and **x**_3_ is the maximum allowable overflow rate (unit: basin·inches/hour) [61]. The model simulates the performance of the given storm drainage system which is defined by the variables, **x**_1_, **x**_2_, and **x**_3_ over a specified period of time and under weather conditions representative of the area.

#### 5.1.2. Objective Functions

There are five objective functions that should be minimized in the WRP problem. The *f*_1_ function is the drainage network cost, *f*_2_ function is the storage facility cost, *f*_3_ function is the treatment facility cost, *f*_4_ function is the expected flood damage cost, and *f*_5_ function is the expected economic loss due to flood. The five objective functions are defined in the following equations:

(6) $$\begin{array}{c} f_{1}\left(x\right)=106780.37\;*\;\left(x_{2}+x_{3}\right)+61704.67,\\ f_{2}\left(x\right)=3000\;*\;x_{1},\\ f_{3}\left(x\right)=\frac{305700\;*\;2289\;*\;x_{2}}{{\left[0.06\;*\;289\right]}^{0.65}},\\ f_{4}\left(x\right)=205\;*\;2289\;*\;e^{-39.75\;*\;x_{2}+9.9x_{3}+2.74},\\ f_{5}\left(x\right)=25\;*\;\left(\frac{1.39}{x_{1}\;*\;x_{2}}+4940\;*\;x_{3}-80\right), \end{array}$$

where **x**_1_, **x**_2_, and **x**_3_ are the three decision variables for the MRP problem.

#### 5.1.3. Constraints

There are seven constraints that should be subjected in the WRP problem. The *g*_1_ function is the average number of floods per year, *g*_2_ function is the average flood volume per year, *g*_3_ function is average number of pounds per year of suspended solids, *g*_4_ function is the average number of pounds per year of settleable solids, *g*_5_ function is the average number of pounds per year of biochemical oxygen demand, *g*_6_ function is the average number of pounds per year of total nitrogen expected flood damage cost, and *g*_7_ function is the average number of pounds per year of orthophosphate. The seven constraints are defined in the following equations:

(7) $$\begin{array}{c} g_{1}\left(x\right)=\frac{0.00139}{x_{1}\;*\;x_{2}}+4.94\;*\;x_{3}-0.08\leq 1.00,\\ g_{2}\left(x\right)=\frac{0.000306}{x_{1}\;*\;x_{2}}+1.082\;*\;x_{3}-0.0986\leq 1.00,\\ g_{3}\left(x\right)=\frac{12.307}{x_{1}\;*\;x_{2}}+49408.24\;*\;x_{3}+4051.02\leq 50000.00,\\ g_{4}\left(x\right)=\frac{2.098}{x_{1}\;*\;x_{2}}+8046.33\;*\;x_{3}-696.71\leq 16000.00,\\ g_{5}\left(x\right)=\frac{2.138}{x_{1}\;*\;x_{2}}+7883.39\;*\;x_{3}-705.04\leq 10000.00,\\ g_{6}\left(x\right)=\frac{0.417}{x_{1}\;*\;x_{2}}+1721.26\;*\;x_{3}-136.54\leq 2000.00,\\ g_{7}\left(x\right)=\frac{0.164}{x_{1}\;*\;x_{2}}+631.13\;*\;x_{3}-54.48\leq 550.00, \end{array}$$

where **x**_1_, **x**_2_, and **x**_3_ are the three decision variables for the MRP problem and 0.01 ≤ **x**_1_ ≤ 0.45, 0.01 ≤ **x**_2_ ≤ 0.10, and 0.01 ≤ **x**_3_ ≤ 0.10.

### 5.2. Walking Fish Group (WFG) Toolkit

The Walking Fish Group (WFG) toolkit [62] is a well-known continuous and combinatorial benchmark suite that can be scaled to any number of objectives and decision variables. Comprised of problems with various characteristics, such as linear, convex, concave, multimodal, disconnected, biased, and degenerated Pareto fronts, the WFG suite is used to challenge varying capabilities of MO algorithms. Their characteristics are summarized in Table 1 [63]. The parameters *k* and *l* in WFG are set to (*m* − 1) and 10, respectively. *m* denotes the number of objectives, and then, the number of variables is set to (*m* − 1) + 10. In this paper, the objective number *m* is set to 2 and 3.

## 6. Experiments and Results Analysis

In this section, we describe the experimental study undertaken to evaluate the performance of the UOF-MOABC framework for solving the water resource planning (WRP) problem and the Walking Fish Group (WFG) toolkit.

### 6.1. Experimental Design

All of the experiments were performed on a PC with the Intel (R) Core i7-4720HQ @ 2.6 GHz 4 cores with 8 GB of RAM with Microsoft Windows 8 Professional Edition Version. We used the Java development kit (JDK) 1.7 and Eclipse 3.2 as the integrated development environment (IDE). And the version of the MOEA framework is 2.12.

#### 6.1.1. Algorithm Selection

We intend to assess the performance of the RMOABC algorithm with six state-of-the-art multiobjective algorithms which were implemented within the MOEA framework, such as the Nondominated Sorted Genetic Algorithm-II (NSGA-II) [5], Nondominated Sorted Genetic Algorithm-III (NSGA-III) [64],Multiobjective *ε*-evolutionary Algorithm Based on *ε* Dominance (*ε*-MOEA) [65], Speed-constrained Multiobjective PSO (SMPSO) [66], Multiobjective Evolutionary Algorithm based on Decomposition (MOEA/D) [67], and the third evolution step of Generalized Differential Evolution (GDE3) [68]. The experiments of simulation were taken on the UOF-MOABC framework which combines the MOEA framework with the RMOABC algorithm in the paper.

NSGA-II algorithm is the second generation of NSGA (Nondominated Sorted Genetic Algorithm) that addressed the deficiencies existing in the construction of nondominated set and the maintenance distribution strategy of a solution set. NSGA-III is the many-objective successor to NSGA-II, using reference points to direct solutions towards a diverse set. *ε*-MOEA utilizes the *ε*-dominance archiving for recording the Pareto optimal solutions; SMPSO is one of the multiobjective PSO algorithms and has the characteristic of limiting the velocity of the particles that will initiate generating the new effective particle positions if the velocity becomes too high. MOEA/D is an optimization algorithm based on the concept of decomposing the problem into many single-objective formulations. GDE3 is a multiobjective differential evolution (DE) algorithm for global optimization with an arbitrary number of objectives and constraints.

#### 6.1.2. Assessment Methods

To allow a quantitative assessment and comparison of the performance of the selected multiobjective optimization algorithms, four indicators, Δ_p_ [57], SP [58], HV [59], and Times were adopted in the experiments. Δ_p_ is an averaged Hausdorff distance composed of GDp [69] and IGDp [69] and measures both diversity and spread [57]. And we take the most typical value *p* = 2 in this paper. The first three indicators are mainly used to evaluate the quality of the obtained Pareto solution set. The last indicator Times is the execution time of the optimization algorithm on the same computer which can reflect the time complexity of computation of the tested algorithm.

#### 6.1.3. Parameter Settings

The selection of algorithm parameters can greatly affect the execution performance; thus, it is generally recommended to fine tune the parameters controlling the searching behavior of the optimization algorithm, such as population size, mutation probability, and crossover probability. Parameter calibration is a very time-consuming and computationally intensive work. Thus, most of the multiobjective algorithms adopted the recommended parameter settings from the MOEA framework in this paper.

The parameter settings of the seven multiobjective algorithms are shown in Table 2. *D* denotes the dimension of decision variables of the optimized problem. The population size is all set to 100, and the external archive capacity is set to 100. The stopping criterion adopted is reaching a certain number of generations, and the maximum evolution number of the algorithm is 10000 for WFG toolkit and is 2500 for WRP problem.

The multiobjective optimization algorithms mentioned above are all the nondeterministic techniques. Thus, it does not make much sense to draw some conclusion after running the algorithm once. The usual solution is to carry out a number of independent runs and then to use means and standard deviations for summarizing the obtained results. Thus, the results obtained from the 30 independent runs of each algorithm were statistically calculated to compare their performance.

### 6.2. Experimental Results for Walking Fish Group (WFG) Problems

#### 6.2.1. Distribution Visualization of the Nondominated Solutions

**Figure 4:**
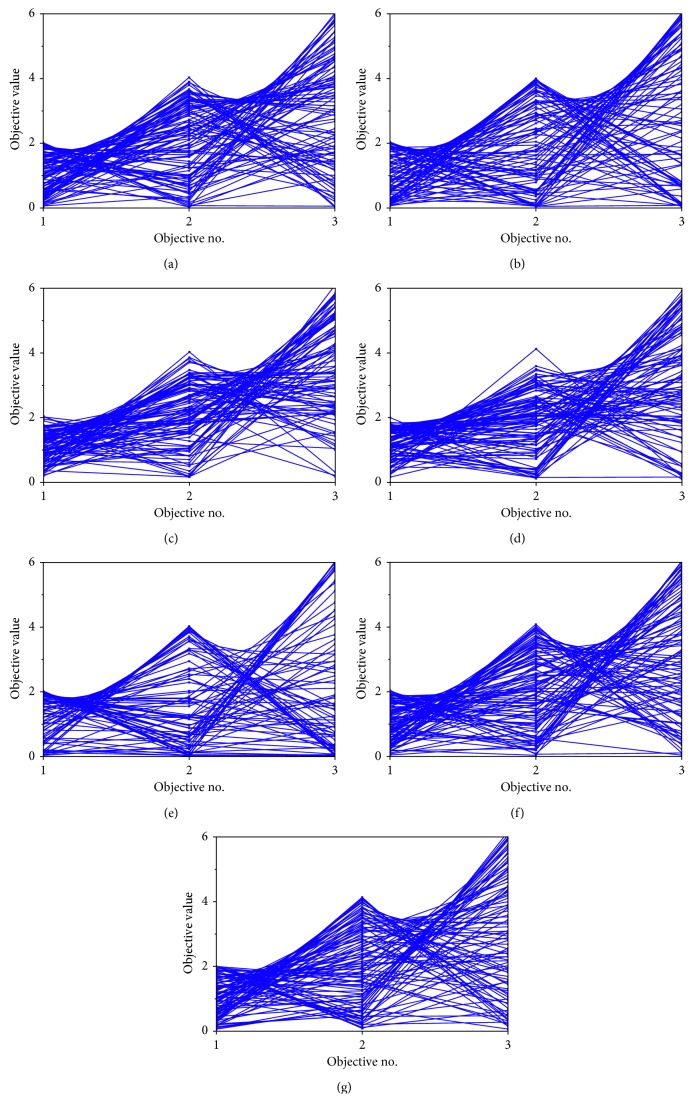
Plots of the final nondominated solution set of the seven algorithms on the 3 objectives of WFG9. (a) NSGA-II algorithm. (b) NSGA-III algorithm. (c) *ε*-MOEA algorithm. (d) SMPSO algorithm. (e) MOEA/D algorithm. (f) GDE3 algorithm. (g) RMOABC algorithm.

To understand the distribution of the nondominated solutions obtained by the seven multiobjective algorithms in the visualization, Figure 4 plots the final nondominated solution set of the seven algorithms on the 3 objectives of WFG9, drawn by the parallel coordinates. Considering the length of the paper, and the WFG9 is the most complicated problem in the WFG test toolkit. Thus, we took it as the case for the study. The experiment result was obtained from the particular run for which its HV value of the result is the closest to the mean value. According to the definitions of the WFG test suite, the upper and lower bounds of the objective *i* of WFG test function are 0 and 2 ∗ *i*, respectively. Thus, the value ranges of objectives for 3 objectives WFG9 are [0, 2], [0, 4], and [0, 6].

Although all the considered nondominated solution sets from the seven algorithms appear to converge into the optimal front, the algorithms perform differently in terms of diversity maintenance, which can be seen from Figure 4. The final nondominated solutions set obtained by NSGA-II, NSGA-III, *ε*-MOEA, SMPSO, and MOEA/D are all not converged in the uniform distribution, and there are many apparent gaps in the range lines of the objective functions, which means the algorithms fail to reach some regions of the Pareto front. The solution sets of GDE3 and RMOABC seem to have a better uniformity and can almost cover all the regions of the Pareto front. And for the NSGA-III, *ε*-MOEA, and GDE3 algorithms, some of their solutions exceed the boundary of the WFG9 problem which can be found in the figures. It can be concluded from the above results that the RMOABC algorithm can converge to the true Pareto front, have a better distribution of nondominated solutions, and appear to have a good covering of the whole Pareto front.

#### 6.2.2. Optimization Process

**Figure 5:**
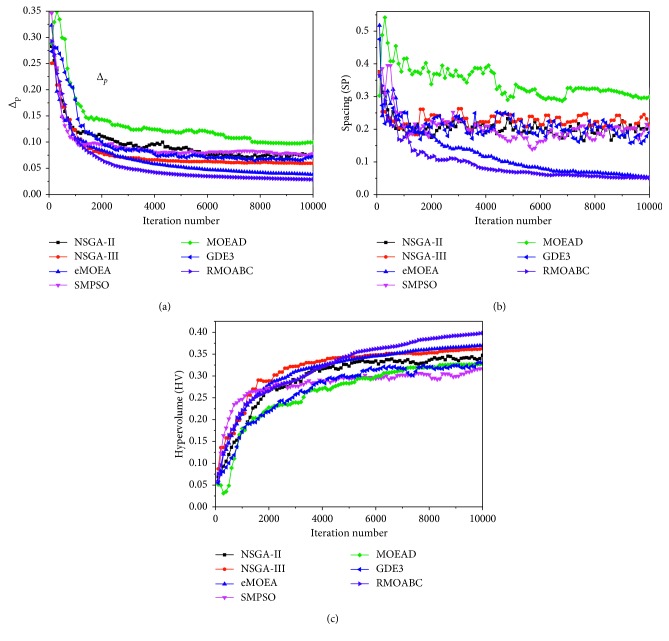
The performance indicators of Δ_p_, SP, and HV vs. the iteration number of the seven multiobjective algorithms for the WFG9 problem with 3 objectives. (a)Δ_p_ indicator. (b) Spacing (SP) indicator. (c) Hypervolume (HV) indicator.

To compare the convergence performance of the seven multiobjective algorithms for the WFG9 problem, the performance indicators of Δ_p_, SP, and HV have been considered as the performance measures in this study. The variations of these three indicators over the iteration number of the seven algorithms are shown in Figure 5. The *X* axis is the iteration number of the algorithms, and the *Y* axis of Figures 5(a)–5(c) represents the values of Δ_p_, SP, and HV of the solutions set of the algorithms, respectively.

It can be seen from the figure, for the Δ_p_ indicator, the seven algorithms perform well in the optimization process, especially the *ε*-MOEA, NSGA-III, and RMOABC algorithm. At about the 1200th iteration, the RMOABC algorithm exceeds other algorithms and outperforms others at the lower Δ_p_ value which means it has a better quality of the solutions. For the SP indicator, the MOEA/D algorithm does not perform well and has a fluctuating line and larger values. The NSGA-II, NSGA-III, SMPSO, and GDE3 perform similarly, and their SP values also fluctuate along the iterations. The *ε*-MOEA and RMOABC have the more stable evolution lines in the SP performance, and the former exceeds the latter at about 1100 iterations and they reach the similar value at about 9500 iterations. For the HV indicator, the NSGA-III, *ε*-MOEA, and RMOABC perform better than other algorithms, especially with the RMOABC algorithm exceeding the others at about 5000 iterations.

#### 6.2.3. Multiobjective Performance Comparison

Through the thirty independent runs of the seven algorithms, the numerical statistical results in terms of the three performance metrics, Δ_p_, SP, and HV and the computational time are shown in Tables 3–10. Max, Min, Mean, and SD represent the maximum value, minimum value, average value, and the standard deviation of the experiments results, respectively. The best mean among the algorithms for each WFG function is shown in bold and italics, respectively.

The statistical results of the Δ_p_ indicator for the WFG problems with 2 objectives are shown in Table 3. A lower value means better computed fronts; thus, we can see that the best or second best indicator values are distributed in three algorithms: NSGA-II, NSGA-III, SMPSO, and RMOABC. NSGA-II, NSGA-III, and SMPSO have computed the best or the second best fronts regarding this indicator in two or three of the evaluated problems; RMOABC has obtained the best or second best values in this indicator for all the problems except the WFG1 and WFG9. For the SP indicator with the WFG problems of 2 objectives in Table 4, RMOABC can obtain the best or second best values in this indicator for all the problems but the WFG3 and WFG5; GDE3 got the best values for WFG5 and WFG7, and *ε*-MOEA got the best value for WFG3. For the HV indicator in Table 5, the larger the value means the better the quality of the solutions. RMOABC can obtain the best and second best values for all the problems but the WFG6; *ε*-MOEA got the best values for WFG5 and WFG6. And for the execution times indicator in Table 6, RMOABC can exceed other algorithms in most of the problems except the WFG1 and WFG2.

The Δ_p_ indicator's statistical results for the WFG problems with 3 objectives are shown in Table 7. It can be seen that the best or second best indicator values are distributed in four algorithms: NSGA-II, NSGA-III, *ε*-MOEA, and RMOABC. RMOABC has computed the best or the second best fronts regarding this indicator in most of the evaluated problems except WFG1 and WFG6. For the SP indicator with the WFG problems of 3 objectives in Table 8, *ε*-MOEA performs also very well in this indicator as it has obtained most of the best values for all the problems; RMOABC can obtain most of the second best values in this indicator for all the problems but the WFG2. For the HV indicator in Table 9, RMOABC can obtain the best and second best values for all the problems but the WFG4 and WFG5; NSGA-II got the best results for WFG3 and WFG4; *ε*-MOEA got the best result for WFG5. And for the execution times indicator in Table 10, RMOABC can exceed other algorithms in most of the problems.

As shown in these tables, most of the algorithms can obtain a good solution set for solving the WFG problems, and the RMOABC and *ε*-MOEA algorithms perform the best for Δ_p_, SP, and HV performance indicators, having a clear advantage over the other five algorithms on most of the test instances of two or three objectives. It demonstrated that the two algorithms are better than the other algorithms in terms of these performance indicators of convergence and distribution. Specifically, RMOABC can also obtain most of the best and second best results of execution times for WFG problems, and *ε*-MOEA needs to take several or even ten times longer to achieve similar results for the 3 objective problems. It demonstrated that the RMOABC can balance the various conflicting objectives and obtain a better performance than other algorithms.

### 6.3. Experimental Results for Water Resources Plan (WRP) Problem

#### 6.3.1. Pareto Optimal Space (Range of Objective Values)

The values range (i.e., the maximum and minimum) of the five objective functions for the water resources planning problem obtained by the seven algorithms is shown in Table 11. As can be seen from the table, there are certain differences between the objective functions' results of the seven algorithms. This is the case because each algorithm has its own way to search the solution space, which may lead to different solutions for achieving similar assessment results.

For easy comparison and calculation, it is necessary to normalize the results of the objective functions. The specific normalized formula is shown in the following equation:

(8) $$\begin{array}{c} X_{\text{normalized}}=\frac{\left(X_{\text{original}}-X_{\min}\right)}{\left(X_{\max}-X_{\min}\right)}, \end{array}$$

where *X*_original_ and *X*_normalized_ are the original values of the objective function and the new value after normalization, respectively and *X*_min_ and *X*_max_ are the minimum and maximum values of the interval of the objective function. These were obtained from the Pareto front file in JMetal multiobjectives optimization framework [15], respectively. Ideally, the values ranges of the objective functions are normalized to [0, 1].

**Figure 6:**
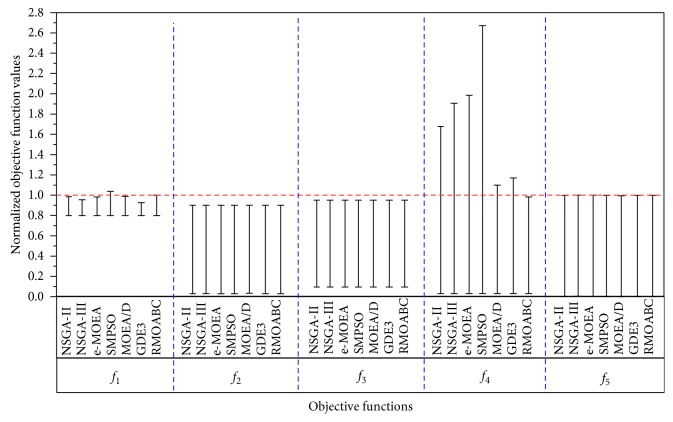
Normalized ranges of the five objective functions' values of the water problem obtained by the seven algorithms.

The high-low-close chart is an efficient graphical tool for visual presentation of various forms of data area such as a range of measured values (min-max), 95% confidence interval value (low limit-high limit), and low value-average value-high value [70]. Thus, the maximum and minimum values of the normalized objectives of Table 11 are presented in Figure 6. The lines indicate the coverage of the objective values for the nondominated solution set obtained by the seven multiobjectives algorithms.

It can be concluded from the figure, the seven algorithms perform better in the objective function *f*_2_, *f*_3_, and *f*_5_ which most of the value space can be covered. But for the objective function *f*_1_, all the seven algorithms can not obtain the better coverage, which they cover the range from 0.8 to 1.0 of the value space. And for the objective function f4, the NSGA-II, NSGA-III, *ε*-MOEA, and SMPSO obtained much larger values than the Pareto front. MOEA/D and GDE3, and especially RMOABC, got better coverage for this objective function.

#### 6.3.2. Optimization Process

**Figure 7:**
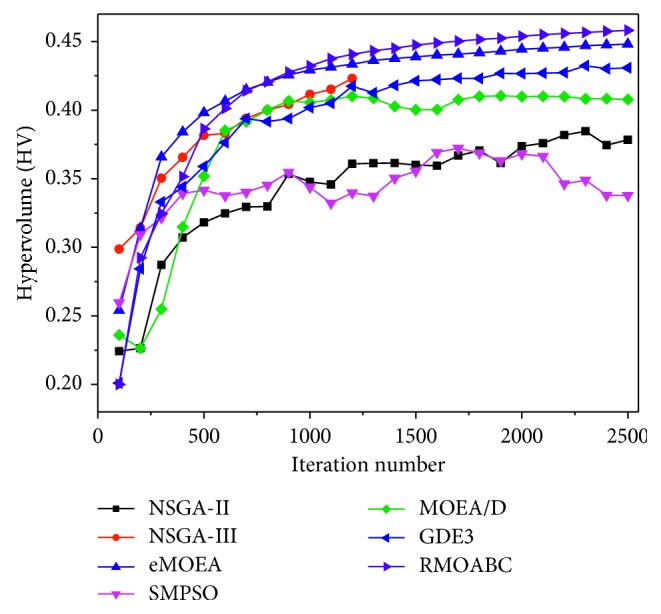
The performance indicator of HV vs. the iteration number of the seven multiobjective algorithms for WRP problem.

Because the WRP problem is a many-objectives (5 objectives) issue and is very complicated, the calculation results of the distribution indicator (spacing) are very high and do not have too many significant representatives. Thus, to compare the performance of the seven multiobjective algorithms for this problem, we took the performance indicator HV as the quality measure in this subsection. The variations of these two indicators over the iteration number of the seven algorithms are shown in Figure 7. The *X* axis is the iteration number of the algorithms, and the *Y* axis represents the values HV of the solutions set of the different algorithms, respectively.

It can be seen from the figures, for the HV indicator, *ε*-MOEA and RMOABC perform better than other algorithms, especially the RMOABC algorithm can exceed the others at about 900 iterations. The other algorithms perform poor, and SMPSO and NSGA-II have a fluctuating line of smaller values.

#### 6.3.3. Multiobjective Performance Comparison

Through the thirty independent runs of the seven algorithms, the performance metric of HV and execution time for the WRP problem are shown in Table 12. The best mean among the algorithms for each WFG function is shown in bold and italics, respectively.

For the HV indicators of the WRP problem, RMOABC obtained the best value, and *ε*-MOEA obtained the second best value. It demonstrated that RMOABC could exceed other algorithms in the performance of convergence and quality for the WRP problem. For the execution times indicator, MOEA/D has the best value, and NSGA-II has the second best value; RMOABC and *ε*-MOEA have to take more time to obtain the final solution set.

## 7. Conclusions

In this paper, we have presented a unified, flexible, configurable, and user-friendly MOABC algorithm framework, UOF-MOABC, which combines a multiobjective ABC algorithm named RMOABC and the multiobjective evolution algorithms (MOEA) framework. Particularly, the core classes of the MOEA framework are described. How to implement the new RMOABC algorithm into the framework has also been illustrated. Then, the Walking Fish Group (WFG) test suite and a many-objective water resource planning (WRP) problem were taken and compared with other six state-of-the-art multiobjective algorithms (NSGA-II, NSGA-III, *ε*-MOEA, SMPSO, MOEA/D, and GDE3) for verification and application.

The obtained experiment results have been statistically analyzed on the basis of three quality indicators (Δ_p_, SP, and HV) with the computation time under the framework. It can be drawn from the experiments that according to the problems, parameter settings, and quality indicators used, the RMOABC method generally outperforms or is equal to the other six MOO algorithms in our study.

As for future work, we plan to integrate more realistic problems to provide more reliable nondominated solution sets for supporting decision making, as well as considering the parallelization of multiobjective ABC algorithms to improve the efficiency and accuracy for solving complex MOPs.

## Figures and Tables

**Algorithm 1:**
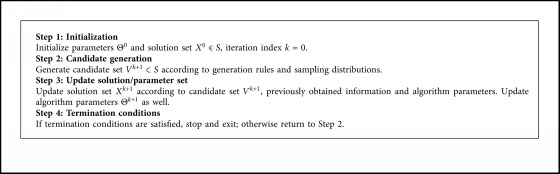
Unity procedure of metaheuristics.

**Table 1:** Properties of the Walking Fish Group (WFG) test problems.

Problem	Number of objectives (*m*)	Number of variables (*n*)	Properties
WFG1	2, 3	(*m* − 1) + 10	Mixed, flat biased
WFG2	2, 3	(*m* − 1) + 10	Convex, disconnected, nonseparable
WFG3	2, 3	(*m* − 1) + 10	Linear, degenerate, nonseparable
WFG4	2, 3	(*m* − 1) + 10	Concave, multimodal
WFG5	2, 3	(*m* − 1) + 10	Concave, deceptive
WFG6	2, 3	(*m* − 1) + 10	Concave, nonseparable
WFG7	2, 3	(*m* − 1) + 10	Concave, parameter dependent biased
WFG8	2, 3	(*m* − 1) + 10	Concave, nonseparable, parameter dependent biased
WFG9	2, 3	(*m* − 1) + 10	Concave, nonseparable, deceptive, parameter dependent biased

**Table 2:** The parameter settings of the seven multiobjective algorithms.

Algorithm	Parameter	Value	Remark
NSGA-II	sbx.rate	0.8	Crossover rate for simulated binary crossover
	sbx.distributionIndex	30.0	Distribution index for simulated binary crossover
	pm.rate	0.2	Mutation rate for polynomial mutation
	pm.distributionIndex	20.0	Distribution index for polynomial mutation
	withReplacement	True	Binary tournament selection

NSGA-III	Divisions	4	Number of divisions
	sbx.rate	0.8	Crossover rate for simulated binary crossover
	sbx.distributionIndex	30.0	Distribution index for simulated binary crossover
	pm.rate	0.2	Mutation rate for polynomial mutation
	pm.distributionIndex	20.0	Distribution index for polynomial mutation

*ε*-MOEA	sbx.rate	0.8	Crossover rate for simulated binary crossover
	sbx.distributionIndex	30.0	Distribution index for simulated binary crossover
	pm.rate	0.2	Mutation rate for polynomial mutation
	pm.distributionIndex	20.0	Distribution index for polynomial mutation
	Epsilon	0.1	*ε* values used by the *ε*-dominance archive

SMPSO	pm.rate	1.0/L	Polynomial mutation.
			*L* = individual length
	pm.distributionIndex	20.0	Distribution index for polynomial mutation

MOEA/D	de.crossoverRate	0.1	Crossover rate for differential evolution
	de.stepSize	0.5	Size of each step for differential evolution
	pm.rate	1.0/L	Mutation rate for polynomial mutation, *L* = individual length
	pm.distributionIndex	20.0	Distribution index for polynomial mutation
	neighborhoodSize	0.1	Neighborhood size used for mating
	Delta	0.9	Probability of mating
	eta	0.01	Maximum number of spots that an offspring can replace

GDE3	de.crossoverRate	0.1	Crossover rate for differential evolution
	de.stepSize	0.5	Size of each step for differential evolution

RMOABC	Adaptive grid number	25	
	*Limit*	0.25∗100∗*D*	*D* denotes the dimension of decision variables

**Table 3:** Statistical results of the Δ_p_ indicator obtained by different algorithms for the WFG problems with 2 objectives.

Functions	Δ_p_	Algorithms						
		NSGA-II	NSGA-III	*ε*-MOEA	SMPSO	MOEA/D	GDE3	RMOABC
WFG1	Max	6.067*E* − 01	7.170*E* − 01	6.473*E* − 01	6.579*E* − 01	4.920*E* − 01	6.225*E* − 01	7.057*E* − 01
	Min	4.795*E* − 01	5.466*E* − 01	6.215*E* − 01	6.133*E* − 01	4.612*E* − 01	5.835*E* − 01	6.362*E* − 01
	Mean	*5.537E − 01*	6.264*E* − 01	6.375*E* − 01	6.400*E* − 01	**4.775*E* − 01**	6.114*E* − 01	6.688*E* − 01
	SD	3.011*E* − 02	4.227*E* − 02	6.966*E* − 03	9.631*E* − 03	7.091*E* − 03	8.040*E* − 03	1.708*E* − 02

WFG2	Max	9.947*E* − 03	3.061*E* − 02	3.249*E* − 02	5.897*E* − 02	2.707*E* − 02	2.044*E* − 02	9.314*E* − 03
	Min	6.454*E* − 03	6.396*E* − 03	1.102*E* − 02	1.910*E* − 02	1.287*E* − 02	1.375*E* − 02	6.167*E* − 03
	Mean	*7.506E − 03*	1.026*E* − 02	2.193*E* − 02	3.474*E* − 02	1.759*E* − 02	1.682*E* − 02	**7.283*E* − 03**
	SD	8.957*E* − 04	5.599*E* − 03	5.465*E* − 03	1.079*E* − 02	3.149*E* − 03	1.771*E* − 03	6.871*E* − 04

WFG3	Max	3.764*E* − 02	3.772*E* − 02	4.460*E* − 02	4.971*E* − 02	3.925*E* − 02	8.769*E* − 01	3.734*E* − 02
	Min	3.469*E* − 02	3.483*E* − 02	3.451*E* − 02	3.941*E* − 02	3.574*E* − 02	7.769*E* − 01	3.412*E* − 02
	Mean	3.607*E* − 02	*3.594E − 02*	3.717*E* − 02	4.221*E* − 02	3.698*E* − 02	8.281*E* − 01	3**.527*E* − 02**
	SD	7.611*E* − 04	6.179*E* − 04	1.819*E* − 03	2.282*E* − 03	7.730*E* − 04	2.367*E* − 02	8.084*E* − 04

WFG4	Max	8.752*E* − 03	7.947*E* − 03	3.409*E* − 02	3.283*E* − 02	1.508*E* − 02	9.889*E* − 03	7.519*E* − 03
	Min	6.084*E* − 03	6.203*E* − 03	2.448*E* − 02	2.718*E* − 02	1.086*E* − 02	7.263*E* − 03	4.554*E* − 03
	Mean	7.622*E* − 03	*6.960E − 03*	2.887*E* − 02	3.073*E* − 02	1.270*E* − 02	8.734*E* − 03	**5.955*E* − 03**
	SD	6.306*E* − 04	4.394*E* − 04	1.916*E* − 03	1.408*E* − 03	1.039*E* − 03	5.439*E* − 04	8.199*E* − 04

WFG5	Max	2.952*E* − 02	2.768*E* − 02	2.805*E* − 02	2.784*E* − 02	2.999*E* − 02	2.723*E* − 02	2.739*E* − 02
	Min	2.794*E* − 02	2.732*E* − 02	2.732*E* − 02	2.721*E* − 02	2.764*E* − 02	2.716*E* − 02	2.725*E* − 02
	Mean	2.845*E* − 02	2.741*E* − 02	2.754*E* − 02	*2.740E − 02*	2.809*E* − 02	2.721*E* − 02	**2.730*E* − 02**
	SD	3.275*E* − 04	9.112*E* − 05	1.708*E* − 04	1.497*E* − 04	4.751*E* − 04	1.662*E* − 05	3.664*E* − 05

WFG6	Max	5.485*E* − 02	5.798*E* − 02	5.974*E* − 02	2.419*E* − 02	3.960*E* − 02	9.971*E* − 02	9.202*E* − 02
	Min	1.786*E* − 02	1.600*E* − 02	1.513*E* − 02	6.189*E* − 03	1.233*E* − 02	7.076*E* − 02	8.184*E* − 03
	Mean	3.583*E* − 02	3.345*E* − 02	3.882*E* − 02	**2.023*E* − 02**	2.614*E* − 02	8.605*E* − 02	*2.194E − 02*
	SD	9.524*E* − 03	8.892*E* − 03	1.230*E* − 02	5.886*E* − 03	6.652*E* − 03	6.550*E* − 03	2.035*E* − 02

WFG7	Max	7.862*E* − 03	7.393*E* − 03	1.028*E* − 02	2.294*E* − 02	9.589*E* − 03	5.893*E* − 03	3.903*E* − 03
	Min	5.856*E* − 03	5.524*E* − 03	5.191*E* − 03	1.100*E* − 02	7.396*E* − 03	4.827*E* − 03	2.797*E* − 03
	Mean	6.632*E* − 03	6.538*E* − 03	6.965*E* − 03	1.639*E* − 02	8.586*E* − 03	*5.352E − 03*	**3.313*E* − 03**
	SD	4.390*E* − 04	4.264*E* − 04	1.292*E* − 03	2.795*E* − 03	6.412*E* − 04	3.028*E* − 04	2.336*E* − 04

WFG8	Max	5.183*E* − 02	5.488*E* − 02	5.574*E* − 02	7.227*E* − 02	5.574*E* − 02	1.034*E* − 01	5.616*E* − 02
	Min	4.772*E* − 02	4.867*E* − 02	5.079*E* − 02	5.286*E* − 02	5.079*E* − 02	9.411*E* − 02	4.580*E* − 02
	Mean	*4.979E − 02*	5.153*E* − 02	5.301*E* − 02	6.299*E* − 02	5.301*E* − 02	9.782*E* − 02	**4.946*E* − 02**
	SD	1.079*E* − 03	1.723*E* − 03	1.282*E* − 03	5.396*E* − 03	1.282*E* − 03	2.444*E* − 03	2.577*E* − 03

WFG9	Max	2.148*E* − 02	1.965*E* − 02	1.017*E* − 01	1.604*E* − 02	1.032*E* − 01	3.266*E* − 02	1.578*E* − 02
	Min	1.006*E* − 02	9.546*E* − 03	7.329*E* − 03	9.397*E* − 03	1.001*E* − 02	9.777*E* − 03	9.608*E* − 03
	Mean	1.533*E* − 02	**1.146*E* − 02**	2.023*E* − 02	*1.177E − 02*	2.293*E* − 02	1.360*E* − 02	1.250*E* − 02
	SD	3.496*E* − 03	2.310*E* − 03	2.780*E* − 02	1.677*E* − 03	2.742*E* − 02	4.199*E* − 03	1.852*E* − 03

**Table 4:** Statistical results of the SP indicator obtained by different algorithms for the WFG problems with 2 objectives.

Functions	SP	Algorithms						
		NSGA-II	NSGA-III	*ε*-MOEA	SMPSO	MOEA/D	GDE3	RMOABC
WFG1	Max	8.933*E* − 01	5.589*E* − 02	4.451*E* − 02	5.944*E* − 01	2.460*E* − 01	8.224*E* − 01	5.282*E* − 02
	Min	6.785*E* − 01	1.363*E* − 02	1.167*E* − 02	4.774*E* − 01	1.988*E* − 01	5.928*E* − 01	1.141*E* − 02
	Mean	7.813*E* − 01	*2.122E − 02*	2.309*E* − 02	5.303*E* − 01	2.304*E* − 01	6.897*E* − 01	**2.048*E* − 02**
	SD	4.920*E* − 02	8.176*E* − 03	8.710*E* − 03	3.291*E* − 02	1.198*E* − 02	5.364*E* − 02	7.507*E* − 03

WFG2	Max	7.803*E* − 02	3.673*E* − 02	3.502*E* − 02	1.057*E* − 01	1.388*E* − 01	6.987*E* − 02	1.788*E* − 02
	Min	3.903*E* − 02	1.929*E* − 02	1.888*E* − 02	3.141*E* − 02	2.923*E* − 02	2.648*E* − 02	1.295*E* − 02
	Mean	5.575*E* − 02	*2.377E − 02*	2.557*E* − 02	5.235*E* − 02	6.459*E* − 02	4.812*E* − 02	**1.549*E* − 02**
	SD	9.663*E* − 03	3.920*E* − 03	4.182*E* − 03	1.830*E* − 02	2.165*E* − 02	1.053*E* − 02	1.376*E* − 03

WFG3	Max	1.498*E* − 02	2.200*E* − 02	1.287*E* − 02	1.803*E* − 02	3.291*E* − 02	1.553*E* − 02	2.317*E* − 02
	Min	8.530*E* − 03	6.070*E* − 03	9.160*E* − 03	9.860*E* − 03	2.222*E* − 02	9.360*E* − 03	1.535*E* − 02
	Mean	1.089*E* − 02	*1.087E − 02*	**1.059*E* − 02**	1.402*E* − 02	2.599*E* − 02	1.209*E* − 02	1.593*E* − 02
	SD	1.481*E* − 03	3.454*E* − 03	9.781*E* − 04	1.970*E* − 03	2.587*E* − 03	1.477*E* − 03	1.637*E* − 03

WFG4	Max	9.755*E* − 02	4.170*E* − 02	4.944*E* − 02	9.454*E* − 02	6.011*E* − 02	6.499*E* − 02	3.345*E* − 02
	Min	4.962*E* − 02	2.205*E* − 02	1.734*E* − 02	2.027*E* − 02	2.269*E* − 02	3.509*E* − 02	1.484*E* − 02
	Mean	7.701*E* − 02	*2.750E − 02*	3.158*E* − 02	5.391*E* − 02	3.457*E* − 02	4.684*E* − 02	**2.180*E* − 02**
	SD	1.044*E* − 02	5.062*E* − 03	7.654*E* − 03	1.792*E* − 02	1.029*E* − 02	7.048*E* − 03	4.040*E* − 03

WFG5	Max	4.109*E* − 02	2.376*E* − 02	2.642*E* − 02	1.835*E* − 02	2.988*E* − 02	1.579*E* − 02	2.551*E* − 02
	Min	2.224*E* − 02	1.860*E* − 02	1.682*E* − 02	9.760*E* − 03	2.394*E* − 02	9.420*E* − 03	1.862*E* − 02
	Mean	3.109*E* − 02	2.029*E* − 02	2.093*E* − 02	*1.323E − 02*	2.603*E* − 02	**1.204*E* − 02**	2.181*E* − 02
	SD	8.089*E* − 03	1.568*E* − 03	2.335*E* − 03	2.001*E* − 03	1.245*E* − 03	1.472*E* − 03	1.960*E* − 03

WFG6	Max	6.226*E* − 02	4.071*E* − 02	4.147*E* − 02	6.487*E* − 02	3.234*E* − 02	7.689*E* − 02	2.759*E* − 02
	Min	2.320*E* − 02	2.259*E* − 02	1.881*E* − 02	1.151*E* − 02	2.260*E* − 02	2.366*E* − 02	1.702*E* − 02
	Mean	4.220*E* − 02	2.753*E* − 02	2.794*E* − 02	*2.259E − 02*	2.596*E* − 02	4.937*E* − 02	**2.063*E* − 02**
	SD	8.941*E* − 03	3.992*E* − 03	6.439*E* − 03	1.310*E* − 02	2.325*E* − 03	1.292*E* − 02	2.721*E* − 03

WFG7	Max	5.291*E* − 02	4.059*E* − 02	3.980*E* − 02	4.875*E* − 02	3.227*E* − 02	3.576*E* − 02	2.591*E* − 02
	Min	2.754*E* − 02	2.158*E* − 02	2.095*E* − 02	1.416*E* − 02	2.328*E* − 02	1.202*E* − 02	1.857*E* − 02
	Mean	3.995*E* − 02	2.645*E* − 02	2.987*E* − 02	2.831*E* − 02	2.672*E* − 02	**2.034*E* − 02**	*2.274E − 02*
	SD	5.877*E* − 03	3.694*E* − 03	4.590*E* − 03	9.755*E* − 03	1.970*E* − 03	5.534*E* − 03	2.023*E* − 03

WFG8	Max	5.703*E* − 02	9.466*E* − 02	5.342*E* − 02	6.918*E* − 02	6.547*E* − 02	9.265*E* − 02	3.139*E* − 02
	Min	3.336*E* − 02	2.278*E* − 02	1.896*E* − 02	1.474*E* − 02	2.526*E* − 02	2.893*E* − 02	1.658*E* − 02
	Mean	4.493*E* − 02	3.385*E* − 02	*2.880E − 02*	3.085*E* − 02	3.604*E* − 02	5.772*E* − 02	**2.145*E* − 02**
	SD	5.287*E* − 03	1.772*E* − 02	8.599*E* − 03	1.032*E* − 02	9.450*E* − 03	1.479*E* − 02	3.257*E* − 03

WFG9	Max	4.272*E* − 02	2.919*E* − 02	3.841*E* − 02	2.287*E* − 02	5.515*E* − 02	2.059*E* − 02	2.072*E* − 02
	Min	1.881*E* − 02	2.045*E* − 02	1.501*E* − 02	1.144*E* − 02	2.463*E* − 02	1.239*E* − 02	1.361*E* − 02
	Mean	2.899*E* − 02	2.347*E* − 02	2.445*E* − 02	*1.586E − 02*	2.831*E* − 02	1.985*E* − 02	**1.522*E* − 02**
	SD	7.041*E* − 03	2.106*E* − 03	5.874*E* − 03	2.602*E* − 03	5.595*E* − 03	2.148*E* − 03	2.303*E* − 03

**Table 5:** Statistical results of the HV indicator obtained by different algorithms for the WFG problems with 2 objectives.

Functions	HV	Algorithms						
		NSGA-II	NSGA-III	*ε*-MOEA	SMPSO	MOEA/D	GDE3	RMOABC
WFG1	Max	1.445*E* − 01	1.049*E* − 01	0.000*E* + 00	0.000*E* + 00	1.391*E* − 01	0.000*E* + 00	1.226*E* − 01
	Min	1.034*E* − 01	5.463*E* − 02	0.000*E* + 00	0.000*E* + 00	1.216*E* − 01	0.000*E* + 00	3.658*E* − 02
	Mean	8.867*E* − 02	8.099*E* − 02	0.000*E* + 00	0.000*E* + 00	**1.306*E* − 01**	0.000*E* + 00	*1.202E − 01*
	SD	1.234*E* − 02	1.457*E* − 02	0.000*E* + 00	0.000*E* + 00	4.708*E* − 03	0.000*E* + 00	1.876*E* − 02

WFG2	Max	5.606*E* − 01	5.599*E* − 01	5.491*E* − 01	5.410*E* − 01	5.545*E* − 01	5.520*E* − 01	5.615*E* − 01
	Min	5.537*E* − 01	5.452*E* − 01	5.260*E* − 01	4.988*E* − 01	5.455*E* − 01	5.378*E* − 01	5.533*E* − 01
	Mean	*5.579E − 01*	5.552*E* − 01	5.385*E* − 01	5.202*E* − 01	5.503*E* − 01	5.438*E* − 01	**5.586*E* − 01**
	SD	1.648*E* − 03	3.042*E* − 03	6.220*E* − 03	1.061*E* − 02	2.209*E* − 03	3.172*E* − 03	1.723*E* − 03

WFG3	Max	4.388*E* − 01	4.387*E* − 01	4.385*E* − 01	4.332*E* − 01	4.378*E* − 01	4.385*E* − 01	4.398*E* − 01
	Min	4.336*E* − 01	4.317*E* − 01	4.320*E* − 01	4.154*E* − 01	4.296*E* − 01	4.344*E* − 01	4.358*E* − 01
	Mean	*4.370E − 01*	4.365*E* − 01	4.352*E* − 01	4.263*E* − 01	4.350*E* − 01	4.359*E* − 01	**4.379*E* − 01**
	SD	1.358*E* − 03	1.413*E* − 03	1.954*E* − 03	3.980*E* − 03	1.504*E* − 03	9.408*E* − 04	1.047*E* − 03

WFG4	Max	2.142*E* − 01	2.153*E* − 01	1.853*E* − 01	1.848*E* − 01	2.082*E* − 01	2.125*E* − 01	2.175*E* − 01
	Min	2.105*E* − 01	2.124*E* − 01	1.777*E* − 01	1.778*E* − 01	1.977*E* − 01	2.076*E* − 01	2.113*E* − 01
	Mean	2.124*E* − 01	*2.138E − 01*	1.821*E* − 01	1.808*E* − 01	2.036*E* − 01	2.097*E* − 01	**2.142*E* − 01**
	SD	9.463*E* − 04	8.231*E* − 04	2.052*E* − 03	1.503*E* − 03	2.283*E* − 03	1.250*E* − 03	1.555*E* − 03

WFG5	Max	1.950*E* − 01	1.955*E* − 01	1.976*E* − 01	1.962*E* − 01	1.945*E* − 01	1.963*E* − 01	1.975*E* − 01
	Min	1.927*E* − 01	1.950*E* − 01	1.971*E* − 01	1.954*E* − 01	1.858*E* − 01	1.961*E* − 01	1.965*E* − 01
	Mean	1.940*E* − 01	1.953*E* − 01	**1.974*E* − 01**	1.959*E* − 01	1.938*E* − 01	1.962*E* − 01	*1.972E − 01*
	SD	5.273*E* − 04	1.348*E* − 04	1.067*E* − 04	2.195*E* − 04	1.554*E* − 03	4.417*E* − 05	2.774*E* − 04

WFG6	Max	1.827*E* − 01	1.917*E* − 01	2.041*E* − 01	2.040*E* − 01	1.883*E* − 01	1.881*E* − 01	1.882*E* − 01
	Min	1.518*E* − 01	1.447*E* − 01	1.153*E* − 01	1.801*E* − 01	1.539*E* − 01	1.608*E* − 01	1.475*E* − 01
	Mean	1.663*E* − 01	1.677*E* − 01	**1.862*E* − 01**	*1.857E − 01*	1.723*E* − 01	1.733*E* − 01	1.679*E* − 01
	SD	9.183*E* − 03	1.006*E* − 02	2.412*E* − 02	6.879*E* − 03	9.347*E* − 03	6.877*E* − 03	1.101*E* − 02

WFG7	Max	2.065*E* − 01	2.069*E* − 01	2.055*E* − 01	1.982*E* − 01	2.036*E* − 01	2.075*E* − 01	2.100*E* − 01
	Min	2.046*E* − 01	2.039*E* − 01	1.989*E* − 01	1.843*E* − 01	2.007*E* − 01	2.057*E* − 01	2.086*E* − 01
	Mean	2.055*E* − 01	2.056*E* − 01	2.031*E* − 01	1.915*E* − 01	2.022*E* − 01	*2.067E − 01*	**2.093*E* − 01**
	SD	5.367*E* − 04	7.907*E* − 04	1.849*E* − 03	3.531*E* − 03	7.302*E* − 04	4.868*E* − 04	3.846*E* − 04

WFG8	Max	1.604*E* − 01	1.599*E* − 01	1.630*E* − 01	1.539*E* − 01	1.589*E* − 01	1.574*E* − 01	1.631*E* − 01
	Min	1.533*E* − 01	1.564*E* − 01	1.479*E* − 01	1.296*E* − 01	1.474*E* − 01	1.534*E* − 01	1.540*E* − 01
	Mean	1.570*E* − 01	*1.582E − 01*	1.551*E* − 01	1.451*E* − 01	1.529*E* − 01	1.558*E* − 01	**1.602E − 01**
	SD	1.546*E* − 03	1.042*E* − 03	3.380*E* − 03	4.554*E* − 03	2.718*E* − 03	1.065*E* − 03	1.703*E* − 03

WFG9	Max	2.308*E* − 01	2.310*E* − 01	2.311*E* − 01	2.322*E* − 01	2.283*E* − 01	2.313*E* − 01	2.341*E* − 01
	Min	2.232*E* − 01	2.251*E* − 01	2.227*E* − 01	2.255*E* − 01	1.252*E* − 01	2.263*E* − 01	1.318*E* − 01
	Mean	2.276*E* − 01	**2.298*E* − 01**	2.178*E* − 01	2.290*E* − 01	2.113*E* − 01	2.287*E* − 01	*2.285E − 01*
	SD	1.840*E* − 03	1.298*E* − 03	2.006*E* − 03	1.548*E* − 03	3.366*E* − 02	1.424*E* − 03	3.450*E* − 02

**Table 6:** Statistical results of the Times indicator obtained by different algorithms for the WFG problems with 2 objectives.

Functions	Times	Algorithms						
		NSGA-II	NSGA-III	*ε*-MOEA	SMPSO	MOEA/D	GDE3	RMOABC
WFG1	Max	3.964*E* + 00	3.767*E* + 00	3.557*E* + 00	3.927*E* + 00	1.841*E* + 00	2.352*E* + 00	3.684*E* + 00
	Min	2.808*E* + 00	2.648*E* + 00	2.492*E* + 00	2.708*E* + 00	1.469*E* + 00	1.741*E* + 00	3.067*E* + 00
	Mean	3.357*E* + 00	3.091*E* + 00	2.912*E* + 00	3.205*E* + 00	**1.642E + 00**	*2.090E + 00*	3.325*E* + 00
	SD	2.148*E* − 01	2.625*E* − 01	2.287*E* − 01	2.992*E* − 01	9.585*E* − 02	1.712*E* − 01	1.466*E* − 01

WFG2	Max	1.071*E* + 00	1.419*E* + 00	1.329*E* + 00	5.720*E* − 01	7.134*E* − 01	6.820*E* − 01	6.424*E* − 01
	Min	8.926*E* − 01	1.130*E* + 00	9.577*E* − 01	3.351*E* − 01	3.878*E* − 01	3.594*E* − 01	4.720*E* − 01
	Mean	9.686*E* − 01	1.269*E* + 00	1.114*E* + 00	**4.264*E* − 01**	5.229*E* − 01	*5.039E − 01*	5.356*E* − 01
	SD	4.895*E* − 02	6.029*E* − 02	1.029*E* − 01	5.993*E* − 02	7.807*E* − 02	7.390*E* − 02	4.307*E* − 02

WFG3	Max	4.544*E* + 00	4.503*E* + 00	8.725*E* + 00	4.700*E* + 00	8.897*E* + 00	4.276*E* + 00	4.383*E* + 00
	Min	4.025*E* + 00	4.255*E* + 00	5.856*E* + 00	4.275*E* + 00	6.634*E* + 00	3.836*E* + 00	3.410*E* + 00
	Mean	4.165*E* + 00	4.356*E* + 00	7.388*E* + 00	4.491*E* + 00	7.981*E* + 00	*4.037E + 00*	**3.724*E* + 00**
	SD	1.304*E* − 01	6.370*E* − 02	5.633*E* − 01	9.786*E* − 02	5.795*E* − 01	1.311*E* − 01	1.934*E* − 01

WFG4	Max	6.624*E* + 00	7.652*E* + 00	1.014*E* + 01	6.290*E* + 00	6.220*E* + 00	7.120*E* + 00	4.619*E* + 00
	Min	6.378*E* + 00	6.721*E* + 00	8.649*E* + 00	4.976*E* + 00	5.096*E* + 00	6.195*E* + 00	3.838*E* + 00
	Mean	6.505*E* + 00	6.922*E* + 00	9.460*E* + 00	5.694*E* + 00	*5.662E + 00*	6.534*E* + 00	**4.238*E* + 00**
	SD	5.756*E* − 02	1.607*E* − 01	3.780*E* − 01	2.880*E* − 01	2.855*E* − 01	2.146*E* − 01	1.624*E* − 01

WFG5	Max	4.160*E* + 00	4.619*E* + 00	8.880*E* + 00	7.595*E* + 00	1.173*E* + 01	1.526*E* + 01	3.993*E* + 00
	Min	3.981*E* + 00	4.462*E* + 00	7.085*E* + 00	3.816*E* + 00	6.699*E* + 00	1.204*E* + 01	3.353*E* + 00
	Mean	*4.093E + 00*	4.536*E* + 00	8.023*E* + 00	4.863*E* + 00	8.559*E* + 00	1.349*E* + 01	**3.773*E* + 00**
	SD	3.828*E* − 02	4.024*E* − 02	4.490*E* − 01	8.183*E* − 01	1.287*E* + 00	7.334*E* − 01	1.685*E* − 01

WFG6	Max	2.158*E* + 00	2.449*E* + 00	4.091*E* + 00	3.982*E* + 00	7.980*E* + 00	1.898*E* + 00	1.675*E* + 00
	Min	1.836*E* + 00	2.139*E* + 00	3.061*E* + 00	2.397*E* + 00	2.307*E* + 00	1.304*E* + 00	1.347*E* + 00
	Mean	2.092*E* + 00	2.336*E* + 00	3.618*E* + 00	3.151*E* + 00	3.688*E* + 00	*1.608E + 00*	**1.528*E* + 00**
	SD	6.471*E* − 02	7.171*E* − 02	2.369*E* − 01	3.580*E* − 01	1.083*E* + 00	1.491*E* − 01	9.100*E* − 02

WFG7	Max	1.169*E* + 01	1.212*E* + 01	2.167*E* + 01	1.209*E* + 01	1.705*E* + 01	2.071*E* + 01	1.022*E* + 01
	Min	1.138*E* + 01	1.183*E* + 01	1.976*E* + 01	1.123*E* + 01	1.499*E* + 01	1.913*E* + 01	9.209*E* + 00
	Mean	*1.153E + 01*	1.194*E* + 01	2.067*E* + 01	1.155*E* + 01	1.596*E* + 01	1.991*E* + 01	**9.607*E* + 00**
	SD	8.228*E* − 02	7.887*E* − 02	4.601*E* − 01	2.109*E* − 01	5.542*E* − 01	4.087*E* − 01	2.124*E* − 01

WFG8	Max	2.152*E* + 00	2.392*E* + 00	3.237*E* + 00	2.500*E* + 00	4.035*E* + 00	1.925*E* + 00	1.623*E* + 00
	Min	1.984*E* + 00	2.226*E* + 00	2.458*E* + 00	2.187*E* + 00	2.925*E* + 00	1.536*E* + 00	1.207*E* + 00
	Mean	2.060*E* + 00	2.319*E* + 00	2.919*E* + 00	2.332*E* + 00	3.541*E* + 00	*1.742E + 00*	**1.373*E* + 00**
	SD	3.506*E* − 02	4.142*E* − 02	1.622*E* − 01	8.102*E* − 02	2.800*E* − 01	9.259*E* − 02	9.533*E* − 02

WFG9	Max	1.333*E* + 01	1.396*E* + 01	3.030*E* + 01	1.329*E* + 01	1.798*E* + 01	1.766*E* + 01	1.366*E* + 01
	Min	1.149*E* + 01	1.206*E* + 01	1.811*E* + 01	1.091*E* + 01	1.392*E* + 01	1.499*E* + 01	9.763*E* + 00
	Mean	1.218*E* + 01	1.266*E* + 01	2.173*E* + 01	*1.213E + 01*	1.632*E* + 01	1.640*E* + 01	**1.075*E* + 01**
	SD	5.191*E* − 01	5.213*E* − 01	3.400*E* + 00	5.491*E* − 01	1.058*E* + 00	6.509*E* − 01	1.113*E* + 00

**Table 7:** Statistical results of the Δ_p_ indicator obtained by different algorithms for the WFG problems with 3 objectives.

Functions	Δ_p_	Algorithms						
		NSGA-II	NSGA-III	*ε*-MOEA	SMPSO	MOEA/D	GDE3	RMOABC
WFG1	Max	5.857*E* − 01	6.439*E* − 01	6.590*E* − 01	6.421*E* − 01	5.322*E* − 01	6.006*E* − 01	7.057*E* − 01
	Min	5.075*E* − 01	5.770*E* − 01	6.170*E* − 01	6.097*E* − 01	5.021*E* − 01	5.523*E* − 01	6.362*E* − 01
	Mean	*5.637E − 01*	6.081*E* − 01	6.440*E* − 01	6.209*E* − 01	**5.169*E* − 01**	5.828*E* − 01	6.688*E* − 01
	SD	1.816*E* − 02	1.526*E* − 02	9.889*E* − 03	7.808*E* − 03	6.408*E* − 03	1.421*E* − 02	1.708*E* − 02

WFG2	Max	7.913*E* − 02	6.738*E* − 02	7.615*E* − 02	1.136*E* − 01	8.929*E* − 02	9.255*E* − 02	4.713*E* − 02
	Min	3.831*E* − 02	4.266*E* − 02	4.438*E* − 02	7.703*E* − 02	5.375*E* − 02	3.868*E* − 02	2.953*E* − 02
	Mean	5.027*E* − 02	*4.897E − 02*	6.064*E* − 02	9.996*E* − 02	6.838*E* − 02	5.455*E* − 02	**3.660*E* − 02**
	SD	9.146*E* − 03	5.690*E* − 03	8.559*E* − 03	1.028*E* − 02	9.356*E* − 03	1.435*E* − 02	4.399*E* − 03

WFG3	Max	8.240*E* − 01	8.902*E* − 01	1.029*E* + 00	8.125*E* − 01	9.034*E* − 01	8.769*E* − 01	8.187*E* − 01
	Min	6.256*E* − 01	6.736*E* − 01	9.144*E* − 01	7.240*E* − 01	7.418*E* − 01	7.769*E* − 01	6.263*E* − 01
	Mean	**7.121*E* − 01**	8.144*E* − 01	9.725*E* − 01	7.594*E* − 01	8.125*E* − 01	8.281*E* − 01	*7.287E − 01*
	SD	4.258*E* − 02	5.841*E* − 02	2.667*E* − 02	2.247*E* − 02	3.910*E* − 02	2.367*E* − 02	5.544*E* − 02

WFG4	Max	8.911*E* − 02	5.596*E* − 02	5.703*E* − 02	8.811*E* − 02	1.343*E* − 01	6.620*E* − 02	4.899*E* − 02
	Min	6.440*E* − 02	5.174*E* − 02	5.025*E* − 02	7.006*E* − 02	8.485*E* − 02	5.248*E* − 02	4.149*E* − 02
	Mean	7.124*E* − 02	*5.370E − 02*	5.391*E* − 02	7.700*E* − 02	1.011*E* − 01	5.989*E* − 02	**4.449*E* − 02**
	SD	5.018*E* − 03	8.658*E* − 04	1.859*E* − 03	4.868*E* − 03	1.431*E* − 02	3.269*E* − 03	1.891*E* − 03

WFG5	Max	8.229*E* − 02	6.334*E* − 02	4.445*E* − 02	8.983*E* − 02	9.643*E* − 02	6.710*E* − 02	6.187*E* − 02
	Min	7.097*E* − 02	5.990*E* − 02	3.336*E* − 02	7.453*E* − 02	7.867*E* − 02	6.120*E* − 02	5.241*E* − 02
	Mean	7.648*E* − 02	6.078*E* − 02	**3.776*E* − 02**	8.087*E* − 02	8.764*E* − 02	6.440*E* − 02	*5.602E − 02*
	SD	2.934*E* − 03	7.096*E* − 04	2.294*E* − 03	3.419*E* − 03	3.822*E* − 03	1.521*E* − 03	2.535*E* − 03

WFG6	Max	1.025*E* − 01	7.740*E* − 02	9.569*E* − 02	1.026*E* − 01	1.362*E* − 01	5.626*E* − 02	8.767*E* − 02
	Min	7.821*E* − 02	6.200*E* − 02	5.607*E* − 02	8.407*E* − 02	9.013*E* − 02	1.846*E* − 02	6.181*E* − 02
	Mean	8.971*E* − 02	*6.938E − 02*	8.187*E* − 02	9.191*E* − 02	1.061*E* − 01	**3.097*E* − 02**	7.806*E* − 02
	SD	6.079*E* − 03	3.611*E* − 03	9.798*E* − 03	4.422*E* − 03	1.071*E* − 02	8.657*E* − 03	6.414*E* − 03

WFG7	Max	7.608*E* − 02	6.288*E* − 02	6.498*E* − 02	1.114*E* − 01	9.321*E* − 02	7.777*E* − 02	4.037*E* − 02
	Min	6.212*E* − 02	5.938*E* − 02	4.303*E* − 02	9.954*E* − 02	7.654*E* − 02	6.489*E* − 02	3.157*E* − 02
	Mean	6.994*E* − 02	6.072*E* − 02	*5.250E − 02*	1.044*E* − 01	8.441*E* − 02	7.151*E* − 02	**3.528*E* − 02**
	SD	3.062*E* − 03	9.551*E* − 04	5.056*E* − 03	3.509*E* − 03	3.443*E* − 03	3.066*E* − 03	2.154*E* − 03

WFG8	Max	5.616*E* − 02	1.027*E* − 01	1.348*E* − 01	1.869*E* − 01	1.360*E* − 01	1.034*E* − 01	9.651*E* − 02
	Min	4.580*E* − 02	8.838*E* − 02	1.069*E* − 01	1.396*E* − 01	1.159*E* − 01	9.411*E* − 02	7.971*E* − 02
	Mean	**4.946*E* − 02**	9.547*E* − 02	1.204*E* − 01	1.602*E* − 01	1.255*E* − 01	9.782*E* − 02	*8.843E − 02*
	SD	2.577*E* − 03	3.067*E* − 03	7.078*E* − 03	1.201*E* − 02	5.490*E* − 03	2.444*E* − 03	4.811*E* − 03

WFG9	Max	1.040*E* − 01	6.317*E* − 02	4.701*E* − 02	8.762*E* − 02	1.204*E* − 01	1.018*E* − 01	5.071*E* − 02
	Min	6.708*E* − 02	5.583*E* − 02	3.034*E* − 02	6.887*E* − 02	7.850*E* − 02	6.713*E* − 02	2.197*E* − 02
	Mean	7.333*E* − 02	5.873*E* − 02	*3.676E − 02*	7.640*E* − 02	9.638*E* − 02	7.550*E* − 02	**3.098*E* − 02**
	SD	6.754*E* − 03	1.407*E* − 03	3.956*E* − 03	3.594*E* − 03	1.000*E* − 02	9.646*E* − 03	6.226*E* − 03

**Table 8:** Statistical results of the *SP* indicator obtained by different algorithms for the WFG problems with 3 objectives.

Functions	SP	Algorithms						
		NSGA-II	NSGA-III	*ε*-MOEA	SMPSO	MOEA/D	GDE3	RMOABC
WFG1	Max	3.085*E* − 01	8.545*E* − 02	4.103*E* − 02	6.136*E* − 01	5.475*E* − 01	6.259*E* − 01	5.051*E* − 01
	Min	5.835*E* − 02	5.663*E* − 02	2.359*E* − 02	5.281*E* − 01	4.439*E* − 01	5.833*E* − 01	3.981*E* − 01
	Mean	8.484*E* − 02	*6.846E − 02*	**3.000*E* − 02**	5.941*E* − 01	5.031*E* − 01	5.998*E* − 01	4.409*E* − 01
	SD	4.410*E* − 02	7.417*E* − 03	4.651*E* − 03	1.582*E* − 02	2.934*E* − 02	1.076*E* − 02	2.246*E* − 02

WFG2	Max	2.708*E* − 01	1.917*E* − 01	1.023*E* − 01	3.927*E* − 01	4.686*E* − 01	3.572*E* − 01	2.003*E* − 01
	Min	1.454*E* − 01	1.296*E* − 01	5.854*E* − 02	1.400*E* − 01	1.142*E* − 01	1.391*E* − 01	9.928*E* − 02
	Mean	1.907*E* − 01	1.593*E* − 01	**7.093*E* − 02**	2.186*E* − 01	1.990*E* − 01	2.084*E* − 01	*1.406E − 01*
	SD	3.311*E* − 02	1.540*E* − 02	1.079*E* − 02	6.788*E* − 02	8.695*E* − 02	5.875*E* − 02	2.625*E* − 02

WFG3	Max	1.526*E* − 01	1.721*E* − 01	4.324*E* − 02	1.585*E* − 01	2.024*E* − 01	1.494*E* − 01	4.979*E* − 02
	Min	9.274*E* − 02	9.738*E* − 02	3.118*E* − 02	1.038*E* − 01	1.464*E* − 01	9.174*E* − 02	3.839*E* − 02
	Mean	1.168*E* − 01	1.267*E* − 01	**3.668*E* − 02**	1.320*E* − 01	1.738*E* − 01	1.236*E* − 01	*4.222E − 02*
	SD	1.313*E* − 02	1.827*E* − 02	2.593*E* − 03	1.273*E* − 02	1.451*E* − 02	1.462*E* − 02	2.523*E* − 03

WFG4	Max	2.560*E* − 01	2.469*E* − 01	5.580*E* − 02	2.942*E* − 01	4.067*E* − 01	2.727*E* − 01	8.951*E* − 02
	Min	1.787*E* − 01	1.974*E* − 01	4.728*E* − 02	1.901*E* − 01	2.618*E* − 01	1.941*E* − 01	5.761*E* − 02
	Mean	2.099*E* − 01	2.236*E* − 01	**5.002*E* − 02**	2.333*E* − 01	3.179*E* − 01	2.248*E* − 01	*6.549E − 02*
	SD	2.243*E* − 02	1.125*E* − 02	2.149*E* − 03	2.309*E* − 02	2.946*E* − 02	2.010*E* − 02	6.137*E* − 03

WFG5	Max	2.791*E* − 01	2.600*E* − 01	5.639*E* − 02	2.443*E* − 01	3.383*E* − 01	2.225*E* − 01	9.423*E* − 02
	Min	1.671*E* − 01	2.073*E* − 01	4.536*E* − 02	1.729*E* − 01	2.644*E* − 01	1.678*E* − 01	5.678*E* − 02
	Mean	2.136*E* − 01	2.313*E* − 01	**5.034*E* − 02**	2.001*E* − 01	2.998*E* − 01	1.920*E* − 01	*7.442E − 02*
	SD	2.163*E* − 02	1.355*E* − 02	2.364*E* − 03	1.876*E* − 02	2.037*E* − 02	1.600*E* − 02	9.141*E* − 03

WFG6	Max	2.782*E* − 01	2.547*E* − 01	6.708*E* − 02	2.605*E* − 01	3.582*E* − 01	2.519*E* − 01	1.034*E* − 01
	Min	1.675*E* − 01	2.123*E* − 01	5.684*E* − 02	1.582*E* − 01	2.657*E* − 01	1.631*E* − 01	7.831*E* − 02
	Mean	2.176*E* − 01	2.265*E* − 01	**6.228*E* − 02**	2.083*E* − 01	3.128*E* − 01	2.080*E* − 01	*8.773E − 02*
	SD	2.555*E* − 02	1.008*E* − 02	3.131*E* − 03	2.524*E* − 02	2.627*E* − 02	2.798*E* − 02	6.767*E* − 03

WFG7	Max	2.704*E* − 01	2.640*E* − 01	5.372*E* − 02	2.963*E* − 01	3.660*E* − 01	3.063*E* − 01	7.872*E* − 02
	Min	1.967*E* − 01	2.201*E* − 01	4.488*E* − 02	1.540*E* − 01	2.533*E* − 01	2.216*E* − 01	5.895*E* − 02
	Mean	2.267*E* − 01	2.383*E* − 01	**4.877*E* − 02**	2.055*E* − 01	3.069*E* − 01	2.499*E* − 01	*6.723E − 02*
	SD	2.023*E* − 02	1.223*E* − 02	2.382*E* − 03	2.483*E* − 02	3.077*E* − 02	2.142*E* − 02	4.472*E* − 03

WFG8	Max	2.633*E* − 01	2.943*E* − 01	6.977*E* − 02	2.517*E* − 01	3.675*E* − 01	2.676*E* − 01	9.905*E* − 02
	Min	1.941*E* − 01	1.941*E* − 01	5.645*E* − 02	1.729*E* − 01	2.549*E* − 01	1.588*E* − 01	6.202*E* − 02
	Mean	2.276*E* − 01	2.493*E* − 01	**6.401*E* − 02**	2.067*E* − 01	3.034*E* − 01	2.196*E* − 01	*7.033E − 02*
	SD	1.842*E* − 02	2.578*E* − 02	3.342*E* − 03	1.658*E* − 02	2.765*E* − 02	2.396*E* − 02	6.679*E* − 03

WFG9	Max	2.485*E* − 01	2.504*E* − 01	5.528*E* − 02	2.419*E* − 01	3.724*E* − 01	2.210*E* − 01	6.936*E* − 02
	Min	1.749*E* − 01	2.003*E* − 01	3.768*E* − 02	1.616*E* − 01	2.634*E* − 01	1.574*E* − 01	4.894*E* − 02
	Mean	2.079*E* − 01	2.258*E* − 01	**4.560*E* − 02**	1.938*E* − 01	3.084*E* − 01	2.021*E* − 01	*5.640E − 02*
	SD	1.865*E* − 02	1.214*E* − 02	3.975*E* − 03	1.819*E* − 02	2.408*E* − 02	1.548*E* − 02	4.673*E* − 03

**Table 9:** Statistical results of the HV indicator obtained by different algorithms for the WFG problems with 3 objectives.

Functions	HV	Algorithms						
		NSGA-II	NSGA-III	*ε*-MOEA	SMPSO	MOEA/D	GDE3	RMOABC
WFG1	Max	1.562*E* − 01	2.069*E* − 01	7.142*E* − 02	7.035*E* − 02	2.499*E* − 01	7.737*E* − 02	2.437*E* − 01
	Min	1.127*E* − 01	1.568*E* − 01	0.000*E* + 00	9.377*E* − 04	2.200*E* − 01	9.640*E* − 03	1.877*E* − 01
	Mean	1.313*E* − 01	1.841*E* − 01	1.748*E* − 02	4.181*E* − 02	**2.339*E* − 01**	4.650*E* − 02	*2.183E − 01*
	SD	1.133*E* − 02	1.323*E* − 02	1.454*E* − 02	1.927*E* − 02	7.751*E* − 03	2.048*E* − 02	1.251*E* − 02

WFG2	Max	8.899*E* − 01	9.022*E* − 01	8.870*E* − 01	8.267*E* − 01	8.835*E* − 01	8.931*E* − 01	9.089*E* − 01
	Min	8.691*E* − 01	8.674*E* − 01	8.511*E* − 01	7.884*E* − 01	8.468*E* − 01	8.769*E* − 01	8.776*E* − 01
	Mean	8.807*E* − 01	*8.912E − 01*	8.698*E* − 01	8.094*E* − 01	8.671*E* − 01	8.860*E* − 01	**8.935*E* − 01**
	SD	6.045*E* − 03	6.742*E* − 03	1.013*E* − 02	1.081*E* − 02	8.704*E* − 03	4.321*E* − 03	7.344*E* − 03

WFG3	Max	2.996*E* − 01	2.812*E* − 01	2.770*E* − 01	2.603*E* − 01	2.183*E* − 01	2.677*E* − 01	2.932*E* − 01
	Min	2.546*E* − 01	2.534*E* − 01	2.298*E* − 01	1.894*E* − 01	1.285*E* − 01	2.268*E* − 01	2.525*E* − 01
	Mean	**2.830*E* − 01**	2.662*E* − 01	2.550*E* − 01	2.233*E* − 01	1.657*E* − 01	2.455*E* − 01	*2.734E − 01*
	SD	1.092*E* − 02	7.071*E* − 03	1.312*E* − 02	1.805*E* − 02	1.857*E* − 02	8.239*E* − 03	1.097*E* − 02

WFG4	Max	3.564*E* − 01	3.877*E* − 01	3.797*E* − 01	3.961*E* − 01	3.558*E* − 01	3.657*E* − 01	3.145*E* − 01
	Min	3.286*E* − 01	3.741*E* − 01	3.598*E* − 01	3.823*E* − 01	3.150*E* − 01	3.475*E* − 01	2.865*E* − 01
	Mean	**3.394*E* − 01**	3.823*E* − 01	3.705*E* − 01	*3.897E − 01*	3.327*E* − 01	3.581*E* − 01	2.981*E* − 01
	SD	6.287*E* − 03	3.306*E* − 03	4.770*E* − 03	3.306*E* − 03	9.216*E* − 03	5.227*E* − 03	6.291*E* − 03

WFG5	Max	3.351*E* − 01	3.729*E* − 01	4.002*E* − 01	3.328*E* − 01	3.254*E* − 01	3.629*E* − 01	3.735*E* − 01
	Min	3.112*E* − 01	3.566*E* − 01	3.823*E* − 01	3.009*E* − 01	3.003*E* − 01	3.436*E* − 01	3.564*E* − 01
	Mean	3.248*E* − 01	*3.676E − 01*	**3.935*E* − 01**	3.179*E* − 01	3.131*E* − 01	3.540*E* − 01	3.638*E* − 01
	SD	6.226*E* − 03	3.928*E* − 03	4.333*E* − 03	9.265*E* − 03	6.171*E* − 03	4.625*E* − 03	4.132*E* − 03

WFG6	Max	3.268*E* − 01	3.595*E* − 01	3.921*E* − 01	3.243*E* − 01	3.314*E* − 01	3.406*E* − 01	3.528*E* − 01
	Min	2.509*E* − 01	2.972*E* − 01	2.980*E* − 01	2.864*E* − 01	2.652*E* − 01	2.740*E* − 01	3.145*E* − 01
	Mean	2.901*E* − 01	*3.327E − 01*	3.236*E* − 01	3.047*E* − 01	2.956*E* − 01	3.086*E* − 01	**3.350*E* − 01**
	SD	1.621*E* − 02	1.656*E* − 02	1.966*E* − 02	8.537*E* − 03	1.653*E* − 02	1.446*E* − 02	9.539*E* − 03

WFG7	Max	3.539*E* − 01	3.787*E* − 01	3.843*E* − 01	2.807*E* − 01	3.444*E* − 01	3.531*E* − 01	4.089*E* − 01
	Min	3.347*E* − 01	3.583*E* − 01	3.561*E* − 01	2.416*E* − 01	3.069*E* − 01	3.270*E* − 01	3.941*E* − 01
	Mean	3.461*E* − 01	*3.722E − 01*	3.708*E* − 01	2.581*E* − 01	3.255*E* − 01	3.380*E* − 01	**4.016*E* − 01**
	SD	5.267*E* − 03	5.421*E* − 03	7.595*E* − 03	8.607*E* − 03	8.995*E* − 03	5.934*E* − 03	3.303*E* − 03

WFG8	Max	2.854*E* − 01	3.056*E* − 01	2.999*E* − 01	2.177*E* − 01	2.653*E* − 01	3.006*E* − 01	3.249*E* − 01
	Min	2.633*E* − 01	2.836*E* − 01	2.674*E* − 01	1.825*E* − 01	2.361*E* − 01	2.816*E* − 01	3.091*E* − 01
	Mean	2.739*E* − 01	*2.936E − 01*	2.818*E* − 01	1.980*E* − 01	2.499*E* − 01	2.910*E* − 01	**3.167*E* − 01**
	SD	5.439*E* − 03	4.792*E* − 03	7.947*E* − 03	8.320*E* − 03	7.743*E* − 03	4.693*E* − 03	5.330*E* − 03

WFG9	Max	3.474*E* − 01	3.746*E* − 01	4.029*E* − 01	3.281*E* − 01	3.396*E* − 01	3.529*E* − 01	4.156*E* − 01
	Min	3.029*E* − 01	3.379*E* − 01	3.702*E* − 01	2.968*E* − 01	2.130*E* − 01	2.491*E* − 01	3.081*E* − 01
	Mean	3.318*E* − 01	3.598*E* − 01	*3.884E − 01*	3.137*E* − 01	3.113*E* − 01	3.296*E* − 01	**3.994*E* − 01**
	SD	1.174*E* − 02	6.881*E* − 03	8.606*E* − 03	8.395*E* − 03	2.881*E* − 02	1.767*E* − 02	1.944*E* − 02

**Table 10:** Statistical results of the Times indicator obtained by different algorithms for the WFG problems with 3 objectives.

Functions	Times	Algorithms						
		NSGA-II	NSGA-III	*ε*-MOEA	SMPSO	MOEA/D	GDE3	RMOABC
WFG1	Max	1.714*E* + 01	2.755*E* + 01	4.232*E* + 01	1.686*E* + 01	1.061*E* + 01	1.648*E* + 01	7.372*E* + 00
	Min	1.321*E* + 01	1.963*E* + 01	3.007*E* + 01	1.474*E* + 01	8.835*E* + 00	1.415*E* + 01	6.949*E* + 00
	Mean	1.405*E* + 01	2.408*E* + 01	3.723*E* + 01	1.591*E* + 01	*9.773E + 00*	1.524*E* + 01	**7.227*E* + 00**
	SD	1.180*E* + 00	2.032*E* + 00	3.331*E* + 00	4.368*E* − 01	3.830*E* − 01	5.030*E* − 01	9.741*E* − 02

WFG2	Max	2.088*E* + 01	4.028*E* + 01	1.032*E* + 02	2.160*E* + 01	1.403*E* + 01	2.099*E* + 01	1.035*E* + 01
	Min	1.816*E* + 01	2.802*E* + 01	7.111*E* + 01	1.830*E* + 01	1.219*E* + 01	1.815*E* + 01	9.545*E* + 00
	Mean	1.877*E* + 01	3.395*E* + 01	8.728*E* + 01	1.956*E* + 01	*1.319E + 01*	1.956*E* + 01	**9.747*E* + 00**
	SD	9.029*E* − 01	2.717*E* + 00	9.289*E* + 00	8.509*E* − 01	5.038*E* − 01	9.577*E* − 01	1.502*E* − 01

WFG3	Max	2.568*E* + 00	4.149*E* + 01	2.535*E* + 02	7.538*E* + 00	1.703*E* + 00	1.353*E* + 01	2.369*E* + 00
	Min	2.002*E* + 00	3.061*E* + 01	1.717*E* + 02	5.360*E* + 00	1.530*E* + 00	1.231*E* + 01	1.820*E* + 00
	Mean	2.179*E* + 00	3.496*E* + 01	2.124*E* + 02	6.428*E* + 00	**1.597*E* + 00**	1.287*E* + 01	*2.006E + 00*
	SD	1.789*E* − 01	2.422*E* + 00	1.673*E* + 01	5.315*E* − 01	4.055*E* − 02	3.292*E* − 01	1.653*E* − 01

WFG4	Max	7.610*E* + 01	6.136*E* + 02	1.013*E* + 03	7.815*E* + 01	4.033*E* + 01	6.893*E* + 01	4.030*E* + 01
	Min	7.086*E* + 01	3.675*E* + 02	7.970*E* + 02	7.291*E* + 01	3.593*E* + 01	6.604*E* + 01	3.468*E* + 01
	Mean	7.251*E* + 01	4.956*E* + 02	9.068*E* + 02	7.569*E* + 01	*3.872E + 01*	6.660*E* + 01	**3.738*E* + 01**
	SD	1.297*E* + 00	4.899*E* + 01	5.167*E* + 01	1.372*E* + 00	1.245*E* + 00	5.100*E* − 01	1.538*E* + 00

WFG5	Max	6.931*E* + 01	4.090*E* + 02	8.132*E* + 02	5.413*E* + 02	4.979*E* + 01	7.366*E* + 01	3.834*E* + 01
	Min	6.487*E* + 01	2.423*E* + 02	6.025*E* + 02	6.403*E* + 01	4.613*E* + 01	7.022*E* + 01	3.257*E* + 01
	Mean	6.702*E* + 01	3.022*E* + 02	6.978*E* + 02	8.082*E* + 01	*4.800E + 01*	7.246*E* + 01	**3.493*E* + 01**
	SD	1.193*E* + 00	4.391*E* + 01	5.262*E* + 01	8.697*E* + 01	1.025*E* + 00	9.243*E* − − 01	1.427*E* + 00

WFG6	Max	6.395*E* + 01	3.368*E* + 02	1.855*E* + 04	6.681*E* + 01	3.925*E* + 01	6.572*E* + 01	3.243*E* + 01
	Min	6.299*E* + 01	2.357*E* + 02	3.978*E* + 02	6.499*E* + 01	3.342*E* + 01	6.437*E* + 01	3.204*E* + 01
	Mean	6.346*E* + 01	2.770*E* + 02	1.081*E* + 03	6.609*E* + 01	*3.640E + 01*	6.496*E* + 01	**3.230*E* + 01**
	SD	1.917*E* − 01	2.562*E* + 01	3.301*E* + 03	4.290*E* − 01	1.401*E* + 00	3.224*E* − 01	8.710*E* − 02

WFG7	Max	6.070*E* + 01	4.729*E* + 02	8.191*E* + 02	6.678*E* + 01	4.132*E* + 01	8.029*E* + 01	3.448*E* + 01
	Min	5.898*E* + 01	3.627*E* + 02	6.596*E* + 02	6.384*E* + 01	3.562*E* + 01	7.275*E* + 01	3.027*E* + 01
	Mean	5.969*E* + 01	3.981*E* + 02	7.463*E* + 02	6.526*E* + 01	*3.875E + 01*	7.508*E* + 01	**3.120*E* + 01**
	SD	4.673*E* − 01	2.470*E* + 01	4.053*E* + 01	7.469*E* − 01	1.268*E* + 00	1.365*E* + 00	8.456*E* − 01

WFG8	Max	6.678*E* + 01	3.988*E* + 03	5.919*E* + 02	7.215*E* + 01	3.747*E* + 01	6.681*E* + 01	3.516*E* + 01
	Min	6.412*E* + 01	3.738*E* + 02	4.801*E* + 02	6.895*E* + 01	3.022*E* + 01	6.565*E* + 01	3.229*E* + 01
	Mean	6.546*E* + 01	5.427*E* + 02	5.325*E* + 02	7.034*E* + 01	*3.447E + 01*	6.643*E* + 01	**3.364*E* + 01**
	SD	6.233*E* − 01	6.511*E* + 02	2.912*E* + 01	8.833*E* − 01	1.722*E* + 00	2.367*E* − 01	8.721*E* − 01

WFG9	Max	6.706*E* + 01	5.353*E* + 02	2.001*E* + 03	6.971*E* + 01	5.869*E* + 01	8.247*E* + 01	3.500*E* + 01
	Min	6.431*E* + 01	2.799*E* + 02	6.042*E* + 02	6.671*E* + 01	4.682*E* + 01	7.644*E* + 01	3.307*E* + 01
	Mean	6.588*E* + 01	4.194E+02	8.709*E* + 02	6.791*E* + 01	*5.213E + 01*	7.941*E* + 01	**3.405*E* + 01**
	SD	6.416*E* − 01	6.430*E* + 01	2.321*E* + 02	6.776*E* − 01	2.355*E* + 00	1.452*E* + 00	6.175*E* − 01

**Table 11:** Values ranges of five objective functions for water resources planning problem obtained by the seven algorithms.

Objective Function	Value Range	Algorithms						
		NSGA-II	NSGA-III	*ε*-MOEA	SMPSO	MOEA/D	GDE3	RMOABC
*f*_1_	Max	78758.58	76429.01	78587.93	83060.74	79119.91	74160.93	79991.59
	Min	63840.31	63842.71	63849.61	63840.28	63840.28	63840.28	63840.28

*f*_2_	Max	1350.00	1349.97	1349.90	1350.00	1350.00	1350.00	1350.00
	Min	41.27	43.74	41.35	43.23	49.97	41.35	41.05

*f*_3_	Max	2853468.42	2852845.28	2853441.68	2853468.96	2853468.96	2853468.96	2853468.96
	Min	285346.90	285392.12	285378.67	285346.90	285346.90	285346.90	285346.90

*f*_4_	Max	10062944.88	11435067.73	11914875.77	16027735.33	6599674.37	7015174.18	5900700.76
	Min	183771.00	184068.53	183942.39	183749.97	183749.97	183749.97	183749.97

*f*_5_	Max	24986.12	24997.07	24997.74	24993.31	24849.60	24989.64	24985.27
	Min	9.26	75.68	45.33	7.22	7.22	7.22	7.22

**Table 12:** Performance comparison of the seven algorithms for water resource plan (WRP) problem.

Indicators		Algorithms						
		NSGA-II	NSGA-III	*ε*-MOEA	SMPSO	MOEA/D	GDE3	RMOABC
HV	Max	4.018*E* − 01	4.314*E* − 01	4.534*E* − 01	3.914*E* − 01	4.148*E* − 01	4.332*E* − 01	4.599*E* − 01
	Min	3.470*E* − 01	4.070*E* − 01	4.388*E* − 01	2.515*E* − 01	2.638*E* − 01	4.259*E* − 01	4.336*E* − 01
	Mean	3.864*E* − 01	4.213*E* − 01	*4.451E − 01*	3.346*E* − 01	3.776*E* − 01	4.301*E* − 01	**4.534*E* − 01**
	SD	1.137*E* − 02	4.885*E* − 03	3.756*E* − 03	3.211*E* − 02	3.887*E* − 02	1.887*E* − 03	4.832*E* − 03

Times	Max	8.137*E* + 00	1.257*E* + 01	1.632*E* + 02	1.098*E* + 01	6.698*E* + 00	1.443*E* + 01	7.555*E* + 01
	Min	7.514*E* + 00	1.049*E* + 01	9.670*E* + 01	9.733*E* + 00	5.790*E* + 00	1.268*E* + 01	2.273*E* + 01
	Mean	*7.774E + 00*	1.115*E* + 01	1.231*E* + 02	1.034*E* + 01	**6.206*E* + 00**	1.340*E* + 01	4.621*E* + 01
	SD	1.505*E* − 01	4.065*E* − 01	1.553*E* + 01	3.475*E* − 01	2.411*E* − 01	3.916*E* − 01	1.221*E* + 01

## Data Availability

The Pareto fronts data used to support the findings of this study are available from the project of jMetal 5.0 which can be downloaded from the web site: https://jmetal.github.io/jMetal/.
